# Fecal microbiota and bile acids in IBD patients undergoing screening for colorectal cancer

**DOI:** 10.1080/19490976.2022.2078620

**Published:** 2022-05-30

**Authors:** Aonghus Lavelle, Stéphane Nancey, Jean-Marie Reimund, David Laharie, Philippe Marteau, Xavier Treton, Matthieu Allez, Xavier Roblin, Georgia Malamut, Cyriane Oeuvray, Nathalie Rolhion, Xavier Dray, Dominique Rainteau, Antonin Lamaziere, Emilie Gauliard, Julien Kirchgesner, Laurent Beaugerie, Philippe Seksik, Laurent Peyrin-Biroulet, Harry Sokol

**Affiliations:** a Gastroenterology Department, Sorbonne University, INSERM, Centre de Recherche Saint-Antoine, CRSA, AP-HP, Saint Antoine Hospital, Paris, France; b Paris Centre for Microbiome Medicine FHU, Paris, France; c APC Microbiome Ireland, University College Cork, Cork, Ireland; d Gastroenterology Department University Claude Bernard Lyon 1Hospices Civils de Lyon, CHU Lyon-Sud, Lyon, France; e Hôpital de Hautpierre, CHU de Strasbourg, Service d’Hépato-gastroentérologie et Assistance Nutritive, Strasbourg, France; f CHU de Bordeaux, Hôpital Haut-Lévêque, Service d’Hépato-gastroentérologie et oncologie digestive – Université de Bordeaux, Bordeaux, France; g Gastroenterology Department, Sorbonne University, INSERM, Centre de Recherche Saint-Antoine, CRSA, AP-HP, Tenon Hospital, Paris, France; h Gastroentérologie, MICI et Assistance Nutritive, DMU DIGEST, hôpital Beaujon, 100 bd du général Leclerc, Clichy, France; i Department of Hepato-Gastroenterology, Hôpital Saint-Louis, Paris, France; j Gastroenterology Department, CHU de Saint-Étienne - Hôpital Bellevue, St Etienne, France; k Gastroenterology Department, Hôpital Européen Georges-Pompidou, Paris, France; l Sorbonne University, Endoscopy Unit, AP-HP, Hôpital Saint-Antoine, Paris, France; m Department of Gastroenterology, Sorbonne Université, INSERM, Institut Pierre Louis d’Epidémiologie et de Santé Publique, Assistance Publique-Hôpitaux de Paris, Hôpital Saint-Antoine, Paris, France; n Department of Gastroenterology, Nancy University Hospital, Nancy, France; o Inserm NGERE, Université de Lorraine, Vandœuvre-Lès-Nancy, France; p FHU Cure, Nancy, France; q INRA, UMR1319 Micalis & AgroParisTech, Jouy en Josas, France

**Keywords:** Gut microbiota, inflammatory bowel disease, dysplasia, colitis-associated colorectal cancer, crohn’s disease, ulcerative colitis, bile acids

## Abstract

Due to the potential role of the gut microbiota and bile acids in the pathogenesis of both inflammatory bowel disease (IBD) and sporadic colorectal cancer, we aimed to determine whether these factors were associated with colorectal cancer in IBD patients. 215 IBD patients and 51 non-IBD control subjects were enrolled from 10 French IBD centers between September 2011 and July 2018. Fecal samples were processed for bacterial 16S rRNA gene sequencing and bile acid profiling. Demographic, clinical, endoscopic, and histological outcomes were recorded. Characteristics of IBD patients included: median age: 41.6 (IQR 22); disease duration 13.2 (13.1); 47% female; 21.9% primary sclerosing cholangitis; 109 patients with Crohn’s disease (CD); 106 patients with ulcerative colitis (UC). The prevalence of cancer was 2.8% (6/215: 1 CD; 5 UC), high-grade dysplasia 3.7% (8/215) and low-grade dysplasia 7.9% (17/215). *Lachnospira* was decreased in IBD patients with cancer, while *Agathobacter* was decreased and *Escherichia-Shigella* increased in UC patients with any neoplasia. Bile acids were not associated with cancer or neoplasia. Unsupervised clustering identified three gut microbiota clusters in IBD patients associated with bile acid composition and clinical features, including a higher risk of neoplasia in UC in two clusters when compared to the third (relative risk (RR) 4.07 (95% CI 1.6–10.3, P < .01) and 3.56 (95% CI 1.4–9.2, P < .01)). In this multicentre observational study, a limited number of taxa were associated with neoplasia and exploratory microbiota clusters co-associated with clinical features, including neoplasia risk in UC. Given the very small number of cancers, the robustness of these findings will require assessment and validation in future studies.

## Introduction

Inflammatory bowel disease (IBD), including Crohn’s disease (CD) and ulcerative colitis (UC), are chronic, relapsing inflammatory conditions of the gastrointestinal tract. Among the many potential factors contributing to morbidity and premature mortality in IBD, the increased risk of developing colorectal cancer is one of the most serious, with IBD patients who have long-standing colonic involvement at risk of developing colitis-associated cancer in addition to sporadic colorectal cancer (CRC).^1^

Colitis-associated cancer and sporadic CRC have both overlapping and distinct features at the molecular level, as well as unique clinical risk factors – notably related to the duration, extent, and severity of colonic inflammation, and the co-existence of primary sclerosing cholangitis (PSC).^1,2^ Recently, there has been interest in the role of the gut microbiota in the pathogenesis of sporadic CRC, particularly the role of *Fusobacterium* species^3,4^ and microbiota-based screening tests have been proposed.^5,6^ The gut microbiota has also been strongly implicated in the pathogenesis of IBD^7,8^ and microbiota-based treatments, such as fecal microbiota transplantation, are being investigated.^9–13^ Furthermore, the gut microbiota has been shown to be important in murine models of colitis-associated cancer.^14,15^ Despite these associations, it remains to be determined whether the gut microbiota may contribute to the development of colitis-associated cancer in human IBD. The potential for gut microbiota-based screening as a noninvasive alternative to colonoscopy in IBD has recently been proposed.^16^

Metabolites produced or transformed by the gut microbiota have also been implicated in the pathogenesis of IBD^17,18^ and of sporadic CRC, especially bile acid metabolites.^19^ Secondary bile acids are produced by the actions of certain colonic bacteria on primary bile acids. Deconjugation of taurine and glycine from primary bile acids is performed by a broad range of species encoding bile salt hydrolases. Taxa expressing the *bai* (bile acid-inducible) operon convert primary bile acids to secondary bile acids and this is performed by a narrow set of Clostridium species (*Clostridium scindens, Clostridium hylemonae* and *Clostridium hiranonis*).^20^ The secondary bile acid deoxycholic acid (DCA), has in particular been associated with sporadic CRC,^19^ while low levels of secondary bile acids have been reported in IBD.^17^

To address whether the gut microbiota and bile acid metabolites were associated with colitis-associated neoplasia (cancer and its precursor lesions, low-grade dysplasia (LGD) and high-grade dysplasia (HGD)), we conducted a multicentre observational study (ClinicalTrials.gov Identifier: NCT02726243) of both the bacterial fecal microbiota by 16S rRNA gene amplicon sequencing and bile acid metabolites in IBD patients undergoing surveillance colonoscopy in France, using non-IBD patients undergoing screening colonoscopy as controls. The aims of this study were to investigate the link between the gut microbiota, intestinal inflammation, colorectal cancer, bile acids, and primary sclerosing cholangitis (PSC). To address these aims, we used both direct comparisons between groups of interest and exploratory, unsupervised Dirichlet Multinomial Mixtures (DMM) to identify microbiome clusters within the data.

## Results

### Study population

**Figure 1.**
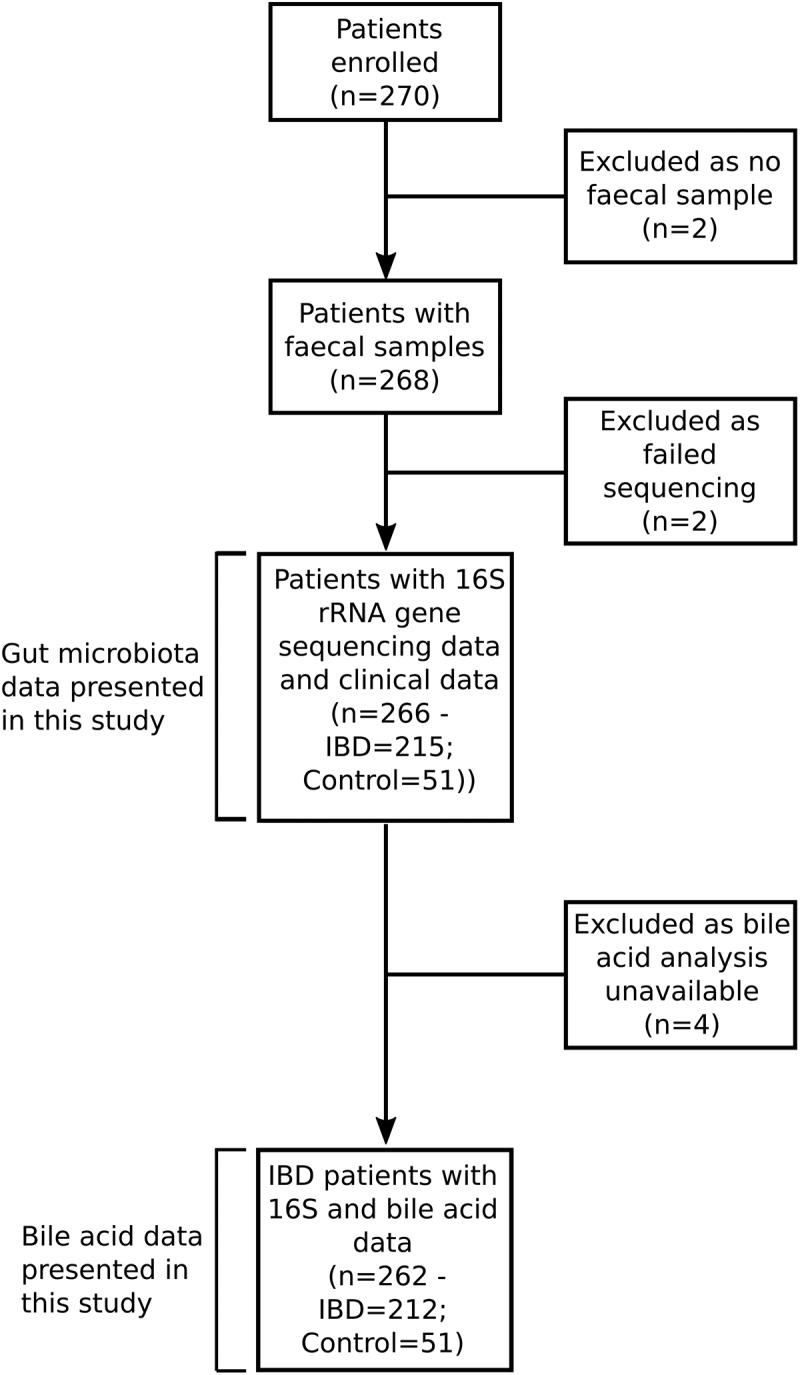
Study flow chart.

**Figure 2.**
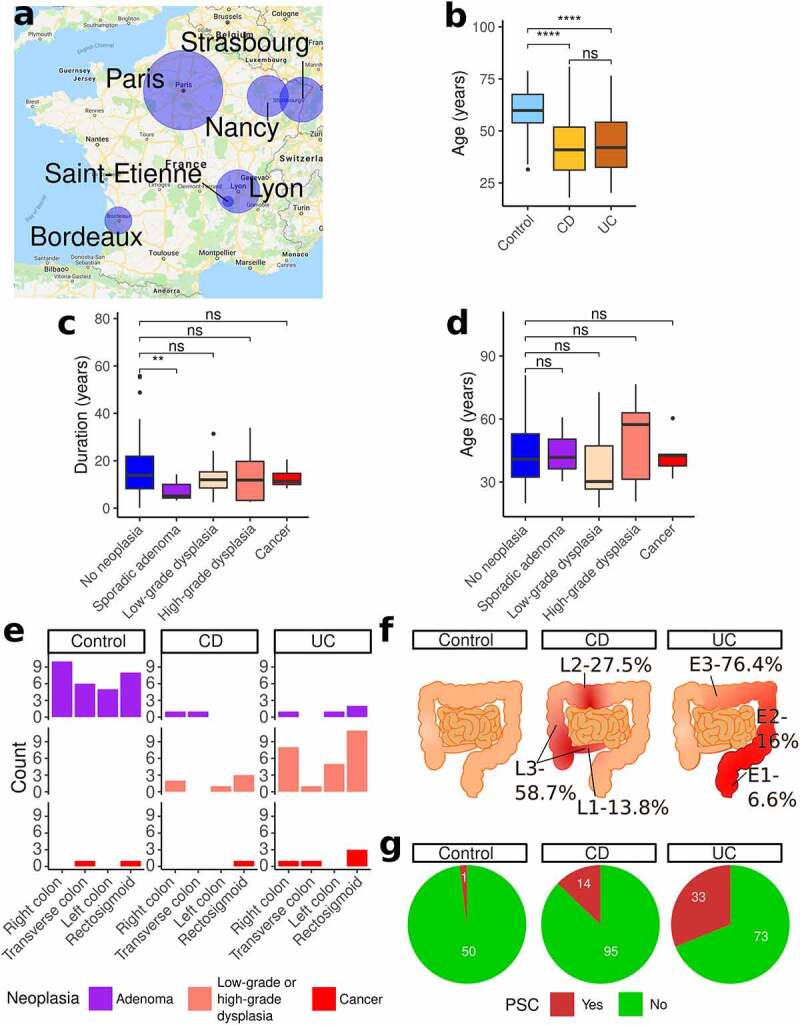
Characteristics of the studied population

A total of 270 patients were recruited into this study from 10 centers in France (Figure 1, Figure 2a and Table S1). Of these, 268 fecal samples were available for sequencing and two samples failed sequencing, leaving 266 patients in the final analysis (106 UC (including undetermined colitis (n = 2)), 109 CD and 51 non-IBD controls (Figure 1, Table S1)). Clinical characteristics are provided in Table 1. Neoplasia was divided into five categories: No neoplasia, sporadic adenomas, low-grade dysplasia, high-grade dysplasia, and colorectal cancer. Two patients had high-grade dysplasia in an adenoma and were included in the high-grade dysplasia category. Patients with more than one finding were classified based on the highest level of neoplasia they exhibited, according to Cancer > high-grade dysplasia > low-grade dysplasia > sporadic adenoma > No neoplasia (Table S2).

**Table 1.** Clinical characteristics of patients recruited to the study. Continuous variables are presented as median (interquartile range). Categorical variables are presented as counts (%). Abbreviations: BMI-Body mass index; HGD-High-grade dysplasia; LGD-Low-grade dysplasia; PPI-Proton pump inhibitors; PSC-Primary sclerosing cholangitis.

Variable	Control	CD	UC	P-value (all)	P-value (CD v UC)
Group [n]	51	109	106		
Age [years]	59.8 (13.7)	40.9 (20.7)	42 (21.7)	p < 1x10^−10^	p = .82
Duration [years]	-	15.2 (14.5)	12 (11.4)		p = .07
Neoplasia None Adenoma LGD HGD Cancer	29 (56.9%) 20 (39.2%) - - 2 (3.9%)	100 (91.7%) 3 (2.8%) 4 (3.7%) 1 (0.9%) 1 (0.9%)	77 (72.6%) 4 (3.8%) 13 (12.3%) 7 (6.6%) 5 (4.7%)		
BMI [kg/m^2^]	25.8 (5.9)	23.4 (5.4)	23.2 (5.4)	p < 5x10^−5^	p = .42
Gender (Female)	28 (54.9%)	53 (48.6%)	48 (45.3%)	p = .53	p = .72
PSC	1 (2%)	14 (12.8%)	33 (31.1%)	p < 1 x10^−5^	p < .01
Intestinal resection	0 (0%)	30 (27.5%)	6 (5.7%)	p < 5x10^−7^	p < 5x10^−5^
Smoke	7 (14%)	24 (22%)	11 (10.4%)	p = .06	p = .03
Antibiotics past 3 months	1 (2%)	11 (10.1%)	6 (5.7%)	p = .15	p = .34
PPI	4 (7.84%)	7 (6.42%)	8 (7.55%)	p = .91	p = .96

a. Map of France with cities contributing to the study and relative contribution in terms of numbers (circle size). b. Age by study group. c. Duration of disease in IBD patients stratified by neoplasia grade. d. Age in years in IBD patients stratified by neoplasia grade. e. Colonic location of adenomas, dysplasia and cancer for each group (note patients can contribute to each location only once but can contribute to multiple locations). f. Disease extent as classified by the Montreal classification. g. Pie charts of proportion of subjects with PSC in each group. CD - Crohn’s disease; IBD - inflammatory bowel disease; PSC - primary sclerosing cholangitis; UC - ulcerative colitis. P-values: * <0.05; ** <0.01; *** <0.001; **** <0.0001.

## Neoplasia detection

Across the three groups, there were eight cancers detected (2/51 (3.9%) non-IBD controls), 1/109 (0.9%) CD patients and 5/106 (4.7%) UC). There were 17 patients with colitis-associated low-grade dysplasia as their highest pathology detected (CD: 4 (3.7%); UC: 13 (12.3%)). In terms of high-grade dysplasia, eight patients had high-grade dysplasia as their highest pathology (CD: 1 (0.9%); UC: 7 (6.6%)), Table 1/Table S2).

Patients with adenomas with low-grade dysplasia (or unclassified) who did not have IBD-associated dysplasia reported separately were classified as sporadic adenomas. These included 20 controls (39.2%), 3 CD (2.8%), and 4 UC patients (3.8%). Finally, 100 CD patients (91.7%), 77 UC patients (72.6%), and 29 controls (56.9%) had no detectable lesions and were classified as having no neoplasia. Table S2 describes the details, including synchronous pathology. Neoplastic colorectal lesions were more common in UC than CD (25 (23.6%) versus 6 (5.5%), p = .0004).

## Cohort characteristics and medication use

Clinical and disease characteristics of IBD patients are presented in Table 2. Oral steroid use, oral, and rectal 5-aminosalicylate (5-ASA) use and tacrolimus use were significantly more common in UC, while the anti-tumor necrosis factor (anti-TNF) medications infliximab and adalimumab were more common in CD. Importantly, IBD patients were significantly younger than non-IBD controls, while non-IBD controls had a significantly higher BMI (Table 1, Figure 2b), limiting direct comparisons between these populations. Disease duration and age across the different categories of neoplasia are presented in Figures 2(c,d), respectively. Neoplasia distribution by colorectal location, disease extent, and PSC are presented in Figures 2(e-g), respectively.

**Table 2.** Disease extent, disease severity and medications specific for IBD patients. Disease extent and medications presented as counts (%). Disease activity presented as median (range). Abbreviations: 5’-ASA-5’-Aminosalicylates; CDEIS-Crohn’s disease endoscopic index of severity; UCEIS-Ulcerative colitis index of severity; UDCA-ursodeoxycholic acid.

	Crohn’s disease (CD)	Ulcerative colitis (UC)	
Montreal (CD) L1 L2 L3 Montreal (UC) E1 E2 E3	15 (13.8%) 30 (27.5%) 64 (58.7%) - - -	- - - 7 (6.7%) 17 (16.2%) 81 (77.1%)	
Harvey-Bradshaw index General well-being Abdominal pain No. liquid stools Abdominal mass Extraintestinal	‘0’ = 85;’1’ = 18;’2’ = 2;’4’ = 1 ‘0’ = 81;’1’ = 16;’2’ = 9;’3’ = 1 Mean = 1.12; SD = 2.02 ‘0’ = 103;’1’ = 2;’2’ = 1 ‘0’ = 80;’1’ = 22	- - - - -	
Partial Mayo score Stool frequency Blood in stool Physician’s global assessment	- - -	‘0’ = 77;’1’ = 19;’2’ = 5;’3’ = 4 ‘0’ = 91;’1’ = 9;’2’ = 4 ‘0’ = 76;’1’ = 11;’2’ = 12;’3’ = 2	
CDEIS	0 (0–17.5)	-	
UCEIS	-	0 (0–8)	
Medications			
Rectal 5’-ASA	3 (2.75%)	12 (11.32%)	p < .05
Oral budesonide	2 (1.83%)	1 (0.94%)	p = 1
Oral steroids	2 (1.83%)	11 (10.38%)	p < .05
Oral 5’-ASA	30 (27.52%)	72 (67.92%)	p < 7e-09
Thiopurine	27 (24.77%)	29 (27.36%)	p = .78
Methotrexate	7 (6.42%)	4 (3.77%)	p = .57
Infliximab	31 (28.44%)	16 (15.09%)	p < .05
Adalimumab	23 (21.1%)	6 (5.66%)	p < .01
Vedolizumab	4 (3.67%)	7 (6.6%)	p = .51
Ustekinumab	1 (0.92%)	0 (0%)	p = 1
Golimumab	2 (1.83%)	3 (2.83%)	p = .68
Mycophenolate	0 (0%)	3 (2.83%)	p = .12
Tacrolimus	0 (0%)	6 (5.66%)	p < .05
UDCA	12 (11.01%)	20 (18.87%)	p = .15

## Significant confounders limited gut microbiota comparisons between IBD patients and non-IBD controls

**Figure 3.**
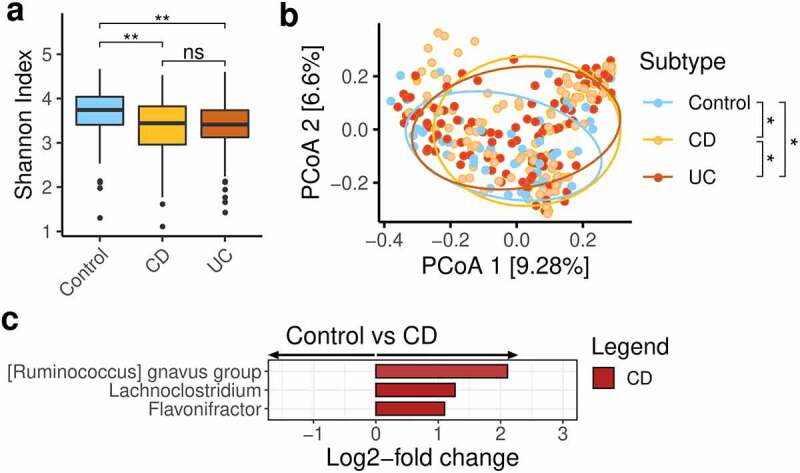
Gut microbiota characteristics of the cohorts in this study

The median number of final sequences per sample was 23823.5 (range 3616–58437). In total there were 6051 ASVs, with 2401 following initial filtering. We first evaluated the study groups to determine if there were differences between non-IBD controls and the different subtypes of IBD. Alpha diversity was reduced in IBD compared to controls overall (non-IBD controls median Shannon index 3.74 (IQR 0.63), CD 3.44 (IQR 0.86), UC 3.41 (IQR 0.61); p = .012, Figure 3a). Beta-diversity analysis suggested differences between control, CD, and UC groups (Figure 3b) although using our consensus approach to differential abundance, only three genera (*Ruminococcus gnavus, Lachnoclostridium* and *Flavonifractor*) were found to be increased in CD versus non-IBD controls (Figure 3c, Figure S1). However, the majority of patients in this study were in clinical remission (175/215–81.4%) and would be expected to have a less altered microbiota.

a. Alpha diversity measures (Shannon diversity index) by study group. b. PCoA plots of proportion-normalised Bray-Curtis divergence, coloured by group with pairwise PERMANOVA significance stars with false-discovery rate applied. c. Differential genera increased in CD versus controls plotted according to their DESeq2 log2-fold change. Genera were only deemed differential when they were detected by all 3 differential abundance metrics employed in this study. CD - Crohn’s disease; PCoA - principle co-ordinate analysis; UC - ulcerative colitis. P-values: * <0.05; ** <0.01; *** <0.001; **** <0.0001.

## *Lachnospira* is decreased in IBD patients with colorectal cancer

**Figure 4.**
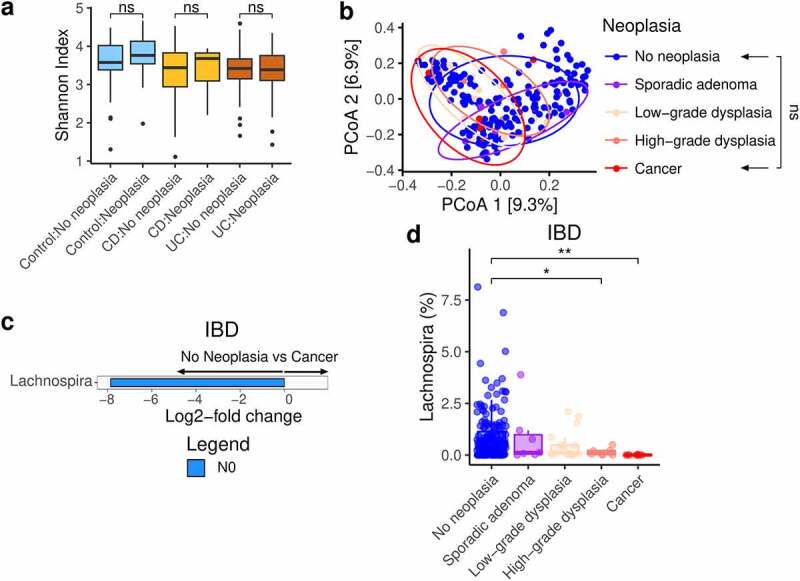
Gut microbiota associated with neoplasia and cancer in IBD

We next looked at neoplasia and compared alpha diversity between patients without neoplasia (N0) and patients with any grade of neoplasia (Nx – sporadic adenoma, low-grade dysplasia, high-grade dysplasia, and cancer) (Figure 4a) and found no significant difference between patients with neoplasia and patients without neoplasia in any of the three cohorts. Despite the low number of IBD-associated cancers, we compared the microbiota between IBD patients without any neoplasia (n = 177) and IBD patients with cancer (n = 6), as this was a primary objective of the study. Pairwise PERMANOVA did not identify a significant difference in community composition (Figure 4b), while differential abundance testing identified only one genus, *Lachnospira*, which was increased in IBD patients without neoplasia compared to IBD patients with cancer (Figure 4c, Figure S2a). Plotting the relative abundance of *Lachnospira* across the different categories of neoplasia confirmed a trend of decreasing *Lachnospira* with more advanced neoplasia in IBD (Figure 4d).

a. Alpha diversity combared between patients with and without neoplasia, stratified by study group. b. PCoA plots of proportion-normalised Bray-Curtis divergence for IBD patients, coloured by category of neoplasia. Pairwise PERMANOVA for patients with any neoplasia and patients with cancer. c. Differential genera increased in IBD patients without cancer versus cancer plotted according to their DESeq2 log2-fold change. Genera were only deemed differential when they were detected by all 3 differential abundance metrics employed in this study. d. Proportion-normalised *Lachnospira* abundance plotted for each category of neoplasia. CD - Crohn’s disease; IBD - inflammatory bowel disease; PCoA - principle co-ordinate analysis; UC - ulcerative colitis. P-values: * <0.05; ** <0.01; *** <0.001; **** <0.0001.

## *Escherichia-Shigella* is increased and *Agathobacter* decreased in UC patients with neoplasia

**Figure 5.**
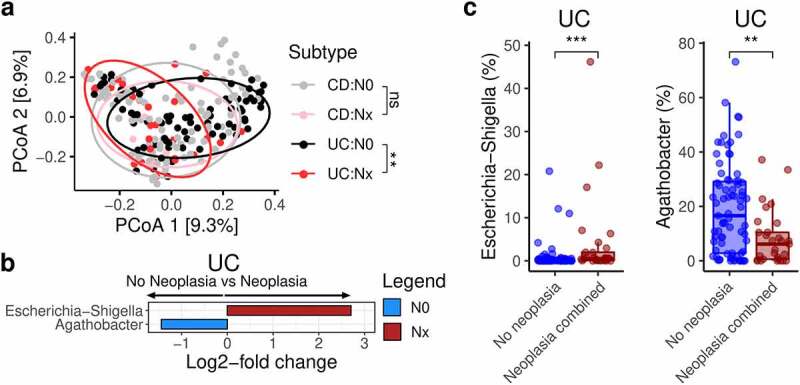
Gut microbiota and neoplasia in ulcerative colitis

We next looked at the combined category of any neoplasia (Nx) versus no neoplasia (N0). No genera were differentially abundant between these two categories for all IBD patients; however, when stratified by subtype, differences were observed in beta diversity in UC patients alone (Figure 5a). Differential abundance testing identified an increase in *Escherichia-Shigella* in UC patients with neoplasia and an increase in *Agathobacter* (formerly *Eubacterium rectale*) in UC patients without neoplasia (Figure S2b, Figure 5(b,c)). Although *Lachnospira* was not significantly increased in N0 UC patients according to DESeq2, it was with the other two methods (Figure S2b and S2c).

a. PCoA plots of proportion-normalised Bray-Curtis divergence for IBD patients, coloured by category of neoplasia. (N0-no neoplasia; Nx - any of sporadic adenoma; low-grade dysplasia; high-grade dysplasia; cancer). b. Differential genera increased in UC patients without neoplasia versus neoplasia plotted according to their DESeq2 log2-fold change. Genera were only deemed differential when they were detected by all 3 differential abundance metrics employed in this study. c. Proportion-normalised *Escherichia-Shigella* and *Agathobacter* abundance plotted for no neoplasia versus neoplpasia combined. CD - Crohn’s disease; IBD - inflammatory bowel disease; PCoA - principle co-ordinate analysis; UC - ulcerative colitis . P-values: * <0.05; ** <0.01; *** <0.001; **** <0.0001.

## Dirichlet multinomial mixtures identify 3 microbiota clusters

Given the exploratory nature of this study and the large number of potentially important co-variates that influence both the microbiota and neoplasia risk, we employed a common unsupervised approach to identifying different community types in microbiome studies, Dirichlet Multinomial Mixtures (DMM). The aim of this approach was to better understand the relationship between different microbiota community types and various clinical variables, including neoplasia.

**Figure 6.**
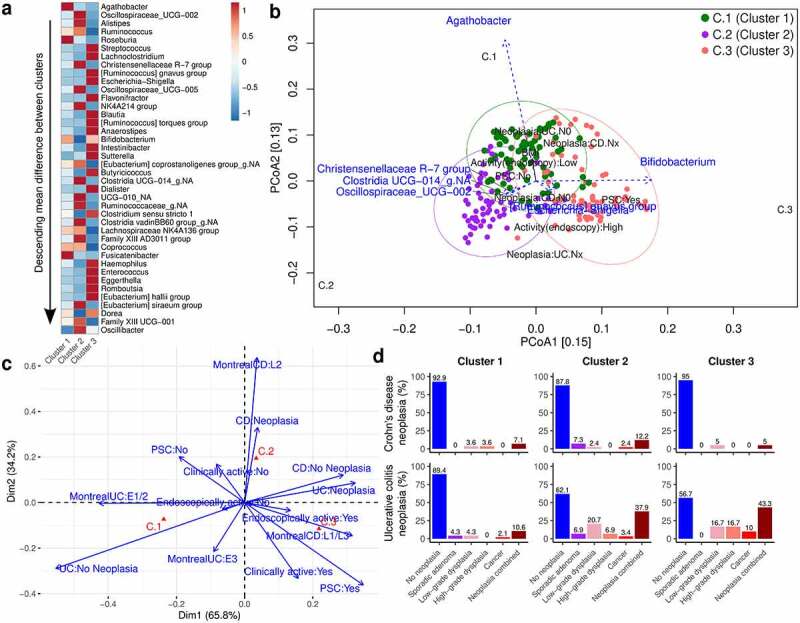
Community types determined by dirichlet multinomial mixtures

Using the Laplace approximation, we determined that the optimum number of clusters was 3 (Figure S3a, with parameters in Figure S3b). Due to inherent variability between runs, we re-fit the model 20 times to ensure convergence (Figure S3c). The top 40 genera that contribute to the cluster assignment are presented in Figure 6a as a row-normalized heatmap of the mean difference in abundance between clusters, descending in order of magnitude. Cluster 1 (C.1) was associated particularly with beneficial butyrate-producing bacteria *Agathobacter* and *Roseburia*, C.2 with *Oscillospiraceae* (UCG-002 and UCG-005), *Alistipes* and *Christensenellaceae* R-7 group and C.3 was associated with a number of pathobionts in IBD, including *Streptococcus, Ruminococcus gnavus* group, and *Escherichia-Shigella* (Figure 6a).

a. Row-normalised heatmap of the top 40 genera influencing DMM cluster composition, in descending order of mean difference between clusters. b. Triplot PCoA of proportion-normalised Bray-Curtis divergence at the genus level, looking at top genera and clinical variables in IBD patients. c. Correspondence analysis plot in IBD patients. d. Plot of the relative proportions of different categories of neoplasia in each cluster, stratified by disease subtype in IBD patients. BMI - body mass index. CD - Crohn’s disease; DMM - Dirichlet multinomial mixtures. IBD - inflammatory bowel disease; PCoA - principle co-ordinate analysis; PSC - primary sclerosing cholangitis. UC - ulcerative colitis.

## Microbiota clusters are associated with clinical characteristics in IBD patients

To visually explore the relationship between the clusters, taxa, and main clinical variables, we used the seqPCoA function to construct a triplot at the genus level (Figure 6b), including the top 10 taxa. Variables included presence of PSC, disease phenotype (Montreal classification), clinical and endoscopic activity (both binary), age, disease duration, body-mass index (BMI), presence of neoplasia stratified by subtype and use of anti-TNF medication, immunomodulators, and 5-ASAs. At q-value threshold of 0.2, 7 taxa and the variables Cluster, neoplasia, PSC, and endoscopic activity were selected. This plot identifies an association between C.1, *Agathobacter*, and UC patients without neoplasia. In contrast, C.3 was associated with *Ruminococcus gnavus* and *Escherichia-Shigella*, PSC and activity on endoscopy. Neoplasia in UC patients was associated with both C.2 and C.3.

To further evaluate the association of DMM clusters and categorical clinical variables, we performed Correspondence Analysis (CA) (Figure 6c) in IBD patients. The variables selected were Neoplasia by subtype, PSC, clinical, and endoscopic activity and disease phenotype. This suggested that C.3 was associated with PSC, clinical and endoscopic activity and CD patients with ileal involvement, while C.1 was associated with UC patients without neoplasia, low endoscopic activity and E1 or E2 disease. Taken together, these findings suggest that C.3 is a traditionally ‘dysbiotic’ cluster and is associated with clinical features such as disease activity, ileal CD and PSC, while C.1 is associated with disease remission, UC patients with more limited disease and UC patients without neoplasia.

Given these observations, we compared the incidence of neoplasia across the three clusters (Figure 6d). No association between neoplasia and microbiome cluster was identified in CD patients (Figure 6d, top panel). In UC (Figure 6d, bottom panel), C.3 had the highest proportion of neoplasia (43.3% – 3 cancers, 5 high-grade dysplasia and 5 low-grade dysplasia out of 30 patients), while 37.9% of UC patients in C.2 had neoplasia (1 cancer, 2 high-grade dysplasia, 6 low-grade dysplasia, 2 sporadic adenomas out of 29). In contrast, only 10.6% of UC patients in C.1 had neoplasia (1 cancer, 2 low-grade dysplasia and 2 sporadic adenomas out of 47). The relative risk of neoplasia in UC C.3 patients compared to C.1 was 4.07 (95% CI 1.6–10.3, p-value = 0.003) and in C.2 compared to C.1 was 3.57 (95% CI 1.4–9.2, p = .009).

## Clinical and technical confounders in the dataset

Consistent with the results of the exploratory triplot and Correspondence analysis (Figures 6(b,c), respectively), 50% of UC patients in C.3 had PSC. In contrast, only 23.4% of patients in C.1 and 24.1% of patients in C.2 had PSC (Chi-squared test P-value = 0.009). As C.3 was associated with other risk factors for neoplasia, such as disease activity and PSC, these may act as confounders. Associations of other important clinical variables with cluster assignment are presented in Table S3.

As described in the methods, a subset of samples (15 out of 215) were fresh-frozen rather than being stored in RNAlater®. While these accounted for a small proportion overall IBD patients (6.97%), they accounted for a large proportion (15/38 (39.5%)) of samples with neoplasia in IBD patients. We compared neoplasia samples fresh-frozen (15) versus neoplasia samples stored in RNAlater (Figure S4). There was no significant difference between the groups by PERMANOVA (Figure S4a), or cluster assignment (Figure S4b), although there is a trend toward increased membership of cluster C.3 in the fresh-frozen samples. Other potential confounders, antibiotic use within the past 3 months (Figure S4c) and sequencing run (Figure S4d) did not appear to have strong effects on composition.

## Bile acids are not associated with cancer or neoplasia but altered between community clusters

**Figure 7.**
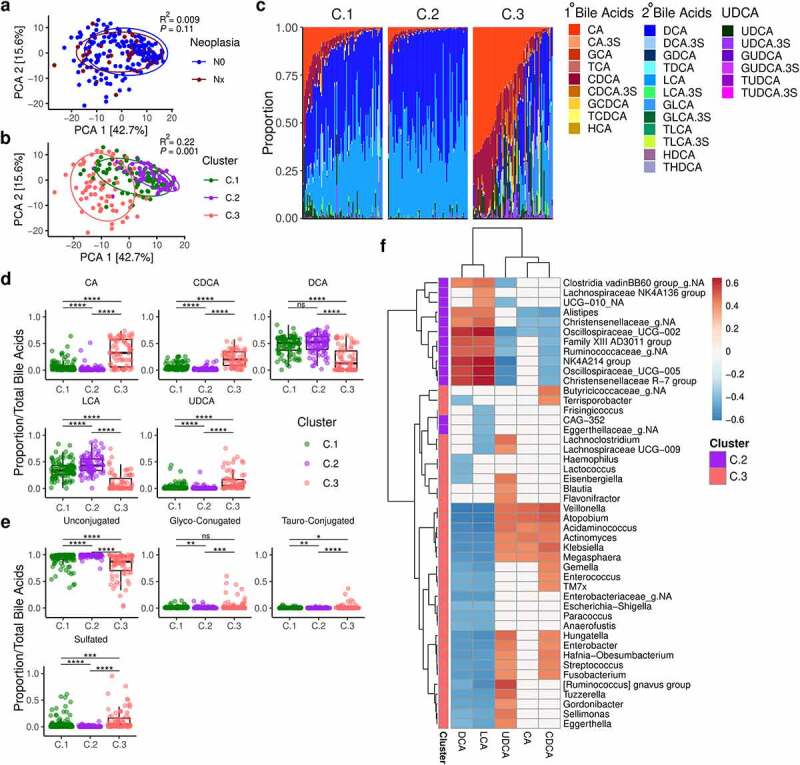
Bile acid composition

Targeted bile acid metabolomics resulted in 27 bile acids, as well as a number of ancillary measurements (Table S4). PCA demonstrated no clear difference between IBD patients with neoplasia and patients without neoplasia and this was confirmed by PERMANOVA testing (p = .11, Figure 7a, Figure S5a). There were no differentially detected bile acids between IBD patients with cancer and those without, nor any difference between IBD patients with and without neoplasia.

a. PCA of bile acids grouped by neoplasia in IBD patients (n=212). b. PCA of bile acids grouped by DMM cluster in IBD patients (n=212). c. Barplots of bile acid in each DMM cluster in IBD patients (n=212). d. Boxplots comparing the proportion of the main 5 bile acids in each cluster across all study participants (n=262). e. Boxplots comparing bile acid conjugation by cluster across all study participants (n=262). f. Heatmap of significant correlations between genera and bile acids (both clr-transformed) across all study participants (n=262). The side-bar indicates the cluster in which the mean abundance of the genus was highest. 1^o^ - primary; 2^o^ - secondary; CA - cholic acid; CDCA - chenodeoxycholic acid; DCA - deoxycholic acid; DMM - Dirichlet multinomial mixtures; LCA - lithocholic acid; N0-no neoplasia; Nx - any grade of neoplasia; PCA - principle component analysis; UDCA - ursodeoxycholic acid. P-values: * <0.05; ** <0.01; *** <0.001; **** <0.0001.

Interestingly, the DMM clusters identified from the microbiota analysis were associated with bile acid composition in IBD patients (PERMANOVA p-value = 0.001, Figure 7b). C.2 and C.3 samples appeared to be at either end of a spectrum with C.1 samples intermediate. This effect appears to be primarily driven by the ratio of primary to secondary bile acids (Figure 7c, Figure S5b). When looking at bile acids across all available patients, the proportion of primary bile acids was highest in C.3 and lowest in C.2, while secondary bile acids were highest in C.2 (Figure 7d). C.2 was also associated with the lowest proportion of conjugated bile acids (Figure 7e, Figure S5c and S5d). When correlating genera with the five main bile acids (CA, CDCA, DCA, LCA, and UDCA), we observed that bacteria that positively correlated with secondary bile acids were most abundant in C.2, while bacteria negatively correlated with secondary bile acids and positively correlated with primary bile acids were most abundant in C.3 (Figure 7f). Due to the limitations of 16S rRNA gene sequencing, we were not able to identify species with known 7α-dehydroxylating capabilities, such as *Clostridium scindens*. While a number of bacteria were correlated positively with secondary bile acids, these are not known to have 7α-dehydroxylating capabilities, although *bai* genes have been reported in uncultured metagenome-assembled genomes closely related to *Oscillospiracea*e,^21^ two members of which (*Oscillospiraceae*-UCG 002 and -UCG 005) correlated positively with DCA and LCA in the current study (Figure 7f). Additionally, *Ruminococcus gnavus* correlated positively with UCDA, consistent with previous reports.^22^

Overall, these data demonstrate that C.3 patients have reduced bile acid deconjugation and primary-to-secondary conversion rates, while C.2 patients have the highest rates. These findings were similar when each study group (control, CD and UC) were analyzed independently (Figure S6). However, CD patients with ileal involvement had a higher proportion of primary bile acids (cholic acid and chenodeoxycholic acid) and a lower proportion of the secondary bile acid lithocholic acid (Figure S7).

## Discussion

Colorectal cancer contributes importantly to the morbidity and premature mortality associated with IBD^1^ and evidence from pre-clinical studies suggests that the microbiota may play a role in tumourigenesis in the setting of inflammation.^15,23–25^ The invasiveness and healthcare costs associated with endoscopic screening mean that risk-based systems, already in place for stratifying surveillance intervals, could be improved by noninvasive biomarkers.

The current study is an exploratory evaluation focusing on the relationships between the gut microbiota, intestinal inflammation, colorectal cancer, bile acid profiles and PSC. This cohort was highly heterogenous in terms of disease subtype, duration, severity, and treatment with a small number of IBD-associated cancers (n = 6), limiting our ability to perform direct comparisons between groups of interest while controlling for confounding variables. Stratification by disease subtype (CD and UC) due to the known differences between these conditions was performed, while neoplasia categories were combined due to the continuous spectrum from low-grade dysplasia to high-grade dysplasia to cancer to increase the power to detect differences. While we have reported a small number of tentative associations by direct comparisons between groups with a consensus differential abundance approach, these are vulnerable to numerous potential confounders for which we were unable to fully control.

To perform a more exploratory approach in this heterogenous cohort, we additionally employed unsupervised microbiome clustering, with the aim of identifying microbiome clusters that were both microbially meaningful in an IBD context and which could be co-associated with clinical variables relevant to CAC. Clusters C.1 and C.3 associated with numerous low-risk and high-risk clinical features, respectively, emphasizing that the gut microbiome may simply be a marker of risk factors for neoplasia, rather than having any direct causal association with its development.

Although the total number of colitis-associated cancers were low (n = 6), differential abundance analysis detected a reduction in *Lachnospira*. This genus is a member of *Lachnospiraceae* and has not been previously associated with CAC. Our differential abundance approach aimed to avoid some of the pitfalls of relying on one method^26^ but as a result, required a relatively high adjusted p-value threshold (0.2) and may also be affected by the highly skewed numbers in each group in this analysis. Given the reduction in *Lachnospira* in high-grade dysplasia as well and the exploratory nature of the study, we have reported this finding, however it should be interpreted with caution and will require validation. Similarly, in UC patients, *Escherichia-Shigella* was increased in neoplasia, while *Agathobacter* was decreased. *Enterobacteriaceae* has been shown to be increased in mucosa of CAC^27^ and are commonly associated with IBD and inflammation. *Escherichia-Shigella* was also prominently associated with C.3, the most dysbiotic cluster associated with the highest prevalence of neoplasia in UC. Targeted editing of the gut microbiota to reduce *E. coli* and commensal *Enterobacteriaceae* has also been shown to reduce colonic tumors in the azoxymethan (AOM)/dextran-sodium sulfate (DSS) model of colitis-associated cancer.^24^ Inflammation appears to independently promote expression of tumor-promoting genes in the *pks* island in *E. col*i.^25^ *Fusobacterium* was not associated with risk of neoplasia in UC patients in this study, consistent with previous results.^27^ Interestingly, while C.3 was the most high-risk cluster for neoplasia in UC patients, this cluster was also associated with ileal disease in CD. Particularly, of 15 patients with isolated ileal disease, 9 patients were in C.3 and only 1 in C.1, consistent with this patient group being characteristically associated with dysbiosis. However, this group had almost no neoplasia (one sporadic adenoma), suggesting that C.3 may represent different things depending on disease subtype. In CD, it may be enriched for patients with ileal CD (not a risk factor for neoplasia), while in UC patients, it may be associated with PSC and disease activity (risk factors for neoplasia).

In contrast, *Agathobacter*, (formerly *Eubacterium rectale*^28^) is a butyrate-producing bacteria that has been shown to be reduced in UC^29,30^ and has been associated with improved response to anti-TNF medications in pediatric IBD.^31^ Butyrate is an important short chain fatty acid produced by fermentation of dietary fiber and has immune-modulating anti-inflammatory and anti-neoplastic effects in the colon.^32^ This genus was strongly associated with C.1. The closely related genus *Roseburia* was also highest in C.1 (Figure 6a), suggesting that these members of the *Lachnospiraceae* may be important for the maintenance of gut health in patients with long-standing ulcerative colitis. DMM models, used as an exploratory technique here, have been used to identify different risk groups for development of atopy in a new birth cohort of infants^33^ and have also been applied to identify microbiota and metabolomic groups in pediatric IBD patients and their relatives.^34^

The mucolytic bacterium *R. gnavus*, closely associated with the high-risk cluster C.3, has already been linked to IBD in multiple studies.^8,35,36^ Interestingly, *R. gnavus* may correlate with active inflammation,^36^ as histological inflammation is strongly associated with colorectal cancer risk in IBD.^37^ While alterations were less clear in C.2, *Alistipes*, which has been associated with sporadic colorectal cancer^38^ as well as mediating colitis-associated cancer risk in Lipocalin-2-deficient/Il10-deficient mice,^39^ was enriched in this cluster (Figure 6a).

In terms of bile acid profiles, C.3 demonstrated marked bile acid dysmetabolism, including increased primary:secondary bile acid ratios, decreased deconjugation and increased sulfation (Figure 6 and Figure S5 and S6). While these findings may be due to a reduction in functional capability of the microbiota, it is also possible that patients in C.3 had a shorter transit time, although we do not have data to support this. C.2, which had the highest relative proportion of secondary bile acids also had a high relative risk of neoplasia compared to C.1. It is interesting to note that while conversion of primary bile acids to secondary bile acids is a hallmark of a functioning colonic microbiota, secondary bile acids have been associated with an increased risk of colorectal cancer.^19^ C.1, the lowest risk cluster, had higher levels of primary bile acids and lower levels of LCA, across the whole cohort (Figure 7d) and in UC and CD patients alone (Figure S6). We note that the differences in bile acid composition across the clusters may be due to other factors, such as diet and may modify the gut microbiota, thus having an indirect effect. However, we did not identify any association with neoplastic risk and bile acid profiles. We hypothesize that other metabolites which were not assessed in this study such as short chain fatty acids and tryptophan metabolites may also be involved, although this will require future studies to investigate.

This study has limitations. Due to its multicentre nature, there may be variability between institutions and providers in terms of approach to surveillance, dysplasia detection, and histopathological assessment, although all centers were reference centers for IBD. Also, a small number of participants would fall outside current surveillance guidelines (15 CD patients with L1 disease and 7 UC patients with E1 disease), while there was a high proportion of patients in the cohort in remission, which may alter microbiota composition and fecal bile acids. Control subjects were also significantly older and had a significantly increased BMI compared to IBD patients (Table 1).

Importantly, there was some heterogeneity in terms of storage as some samples were fresh-frozen as opposed to being stored in RNAlater®, which may be a source of bias. In addition, 16S rRNA gene sequencing for this study was performed over two separate sequencing runs, one 250bp paired-end and the other 300 bp paired-end sequencing. To account for this, we performed separate error-learning steps with the dada2 algorithm and ensured identical trimming lengths.

A number of potentially important predictor variables were not available. These include family history of colorectal cancer, previous dysplasia, and post-inflammatory polyps, although the utility of the latter has recently been brought into question.^40^ Finally, missing data prevented us from providing a full analysis of the endoscopic disease activity scores, as these were incomplete, absent or incorrectly applied in 10 IBD patients. There was also some missing data in relation to the clinical conditions described in Table 1 (Spondyloarthropathies, Appendicectomy, Psoriasis).

## Conclusion

In this multicentre study of the fecal microbiota and bile acid profiles of IBD patients undergoing colorectal cancer surveillance in France, we identified a small number of taxa and high- and low-risk community clusters associated with neoplasia in UC. These microbiota changes were closely associated with other high-risk features, such as inflammation and PSC and whether they are markers of high-risk disease or have causal link to neoplasia will require mechanistic studies. These findings will also require future validation in large, prospective cohort studies.

## Methods

### Ethical approval

Approval for human studies was obtained from the local ethics committee (Comité de Protection des Personnes Ile-de-France IV, IRB 00003835 Dyscolic study; registration number 2014/10NICB). Patients or the public were not involved in the study design.

## Inclusion and exclusion criteria

For inclusion, patients must be 18 years of age or older, have the capacity to give informed consent, have IBD or be a non-IBD control undergoing a scheduled screening colonoscopy, diagnoses confirmed in any of the participating service according to ECCO consensus and patient to have follow-up in one of the participating services. Exclusion criteria were trusteeship, guardianship or safeguard justice, unable to speak French, answer questions or speak, history of colonic resection, ‘ostomy’ at time of colonoscopy and current treatment by radiotherapy or chemotherapy. Patients who had received antibiotics within the past 3 months were included, although this was initially a temporary exclusion criteria.

## Recruitment

Adult IBD patients undergoing surveillance colonoscopy were recruited in 10 French IBD centers (Figure 2a, Table S1) and provided informed consent. Non-IBD adult patients undergoing screening colonoscopy for CRC were identified in routine clinical practice. Demographic and clinical details were recorded. Clinical activity was also assessed using the partial Mayo score (for UC) and the Harvey-Bradshaw index (for CD).

## Colonoscopy

Colonoscopy was performed according to local protocols at each institution. Endoscopy reports and histological outcomes were provided.

## Fecal sample collection

Patients who consented to participate provided a single fecal sample which was stored in RNAlater® prior to colonoscopy and bowel preparation. A subset of IBD samples (15 out of 215) were fresh-frozen rather than being stored in RNAlater®. While these accounted for a small proportion of IBD patients overall (6.97%), they accounted for a large proportion (15/38 (39.5%)) of samples with neoplasia in IBD patients. Fecal samples were then transferred to the central receiving laboratory at the Center de Recherche Saint Antoine (CRSA), Paris.

## Microbial DNA extraction

DNA was extracted from fecal samples by both mechanical and chemical methods, as previously described.^41^ Microbial lysis was performed by both mechanical and chemical methods. Briefly, mechanical lysis was performed with glass beads and following isopropanol precipitation of nucleic acids for 10 min at room temperature, samples were incubated on ice for 15 min and then centrifuged for 30 min at 20 000 g and 4°C. The resulting pellets were suspended in phosphate buffer (450 μL) and potassium acetate (50 μL). Following RNase treatment and DNA precipitation, recovery of nucleic acids was performed via centrifugation at 20 000 g and 4°C for 30 min. The DNA pellet was suspended in 80 μL of trypsin-EDTA buffer.

## 16S rRNA gene amplicon sequencing

Amplicon sequencing of the V3-V4 region of the 16S ribosomal RNA gene was employed for microbiota analysis. The primers used for this analysis were – 16S sense 5′-TACGGRAGGCAGCAG-3′ and anti-sense 5′-CTACCNGGGTATCTAAT-3′. This was performed using an optimized and standardized 16S amplicon library preparation protocol (Metabiote, GenoScreen, Lille, France). 16S DNA PCR was performed with 5ng of genomic DNA with bar-coded primers (Metabiote MiSeq Primers) according to the manufacturer’s protocol (Metabiote) at a final concentration of 0.2 μmol/L, with an annealing temperature of 50°C for 30 cycles. PCR product purification was performed with Agencourt AMPure XP-PCR purification system (Beckman Coulter, Brea, CA, USA) and was quantified according to the manufacturer’s protocol with samples multiplexed at equal concentrations. An Illumina MiSeq platform (Illumina, San Diego, CA, USA) was used for sequencing and this was performed over two separate sequencing runs: a 250 bp paired-end sequencing protocol and a 300 bp paired-end sequencing protocol, at GenoScreen. Raw paired-end sequencing reads were subjected to the following initial procedures a GenoScreen: (1) quality filtering with the PRINSEQ-lite PERL script,^42^ truncating bases from the 3′ end with a quality <30 (based on the Phred algorithm) and (2) using CutAdapt to remove primers, with no mismatches allowed in the primer sequences.^43^ Only sequences with perfectly matching forward and reverse primers were retained for further analysis.

## 16S rRNA gene sequence analysis

Amplicon sequence variants (ASVs) were determined using the dada2 algorithm,^44^ applied independently per sequencing run. Taxonomic classification was performed using the Silva reference database (version 138).^45^ R scripts are available on github (https://github.com/ajlavelle/dyscolic-Figures).

Data was then imported into the R statistical environment for subsequent analysis (R version 3.6.3,^46^ *phyloseq* package (version 1.28.0),^47^ incorporating the Bioconductor workflow)^[48]^ 16S rRNA gene sequence data are deposited in the Sequence Read Archive (accession number PRJNA720094). Bile acid metabolomics data and selected metadata are available from the corresponding author on request (harry.sokol@gmail.com).

## Targeted bile acid metabolomics

Bile acid metabolomics were performed as previously described.^17^

## Statistical and microbiome analysis

Continuous data is presented as median and inter-quartile range (IQR). Between-group differences were assessed for continuous data using the Wilcoxon rank sum test for two groups and the Kruskal-Wallis test for >2 groups and for categorical data using the Chi-squared test or the Fisher exact test. P-values are represented with stars according to the following convention: * <0.05; ** <0.01; *** <0.001; **** <0.0001. P-values plotted by *ggpubr* are uncorrected.

Filtering of microbiota data was performed initially to remove ASVs that were not assigned to a Phylum or which were present on only a single individual. Microbiota alpha diversity was estimated using the Shannon diversity index. Total-sum scaled (TSS) normalized data was generated for beta-diversity assessment using the Bray-Curtis index. PERMANOVA was performed using the adonis function with 999 permutations (*vegan* package,^49^ version 2.5–6). Differential abundance between groups was tested at the genus level in genera present in at least 10% of individuals using an ensemble or consensus approach, as suggested by a recent publication.^26^ This included a standard Wilcoxon rank sum test on TSS normalized data, an approach using centered-log ratio (clr) transformed data as implemented in the ALDEx2 package,^50^ the Wilcoxon test on total sum scaled proportions and a negative binomial count method implemented in the *DESeq2* package (version 1.24.0).^51^ A false discovery rate threshold of 0.2 was applied and only taxa which were detected concordantly by all three approaches were included and plotted based on their DESeq2 ‘log2foldchange’. Plotting was performed using *ggplot2* (version 3.2.1)^52^ and *ggpubr* (version 0.2.3).^53^ Triplots were created using the ‘seqPCoA’ function from the *seqgroup* package (https://github.com/hallucigenia-sparsa/seqgroup/) which wraps PCoA, PERMANOVA and envfit functions from the *vegan* package.

Dirichlet multinomial mixture (DMM) clustering of bacterial genera was performed with the *DirichletMultinomial* package in R (version 1.26.0),^54,55^ using the number of clusters that minimized the Laplace approximation. Due to variability between runs, this procedure was re-run 20 times to ensure a convergent result.

For bile acid metabolites, TSS normalized data was compared between groups. For principal component analysis (PCA), bile acids concentrations were normalized and transformed in a similar manner as used in the *metaboanalystR* package (version 3.0.3),^56^ replacing undetected values with 1/5^th^ of the minimum value per feature, followed by log-2 transformation and mean centering. ALDEx2 was used to test for differentially abundant bile acid metabolites.

The relative risk of dysplasia between low-risk and high-risk clusters was calculated with 95% confidence intervals^57^ and statistical significance tests were performed using the Fisher exact test. For correlation between bacterial genera and bile acid metabolites (cholic acid (CA), chenodeoxycholic acid (CDCA), deoxycholic acid (DCA), lithocholic acid (LCA) and ursodeoxycholic acid (UDCA)), zero values were imputed using the ‘cmult’ function from the *zCompositions* package,^58^ followed by the clr transformation from the *compositions* package.^59^ A Spearman rank correlation was performed using the ‘rcorr’ function from the *Hmisc* package and only values with an FDR-corrected p-value of <0.2 and an absolute correlation coefficient of >0.4 were included. Genera were assigned to the DMM cluster for which the had the highest mean abundance and the resulting heatmap was plotted using the *pheatmap* package.

## Supplementary Material

Supplemental Material

## References

[1] Beaugerie L, Itzkowitz SH, Longo DL. Cancers complicating inflammatory bowel disease. New England J Med. 2015;372(15):1441–18. doi:10.1056/NEJMra1403718. 25853748

[2] Kornfeld D, Ekbom A, Ihre T. Is there an excess risk for colorectal cancer in patients with ulcerative colitis and concomitant primary sclerosing cholangitis? A population based study. Gut. 1997;41(4):522–525. doi:10.1136/gut.41.4.522. 9391253 PMC1891549

[3] Kostic AD, Chun E, Robertson L, Glickman J, Gallini C, Michaud M, Clancy T, Chung D, Lochhead P, Hold G, *et al*. Fusobacterium nucleatum potentiates intestinal tumorigenesis and modulates the tumor-immune microenvironment. Cell Host Microbe. 2013;14(2):207–215. doi:10.1016/j.chom.2013.07.007. 23954159 PMC3772512

[4] Bullman S, Pedamallu CS, Sicinska E, Clancy TE, Zhang X, Cai D, Neuberg D, Huang K, Guevara F, Nelson T, *et al*. Analysis of Fusobacterium persistence and antibiotic response in colorectal cancer. Science (1979). 2017;358:1443–1448. doi:10.1126/science.aal5240. PMC5823247 29170280

[5] Yu J, Feng Q, Wong SH, Zhang D, Liang QY, Qin Y, Tang L, Zhao H, Stenvang J, Li Y, *et al*. Metagenomic analysis of faecal microbiome as a tool towards targeted non-invasive biomarkers for colorectal cancer. Gut. 2017;66(1):70–78. doi:10.1136/gutjnl-2015-309800. 26408641

[6] Flemer B, Warren RD, Barrett MP, Cisek K, Das A, Jeffery IB, Hurley E, O'Riordain M, Shanahan F, O'Toole PW, *et al*. The oral microbiota in colorectal cancer is distinctive and predictive. Gut. 2017. Published Online First. doi:10.1136/gutjnl-2017-314814. PMC6204958 28988196

[7] Sokol H, Pigneur B, Watterlot L, Lakhdari O, Bermúdez-Humarán LG, Gratadoux JJ, Blugeon S, Bridonneau C, Furet JP, Corthie, G, *et al*. Faecalibacterium prausnitzii is an anti-inflammatory commensal bacterium identified by gut microbiota analysis of Crohn disease patients. Proc Natl Acad Sci U S A. 2008;105(43):16731–16736. doi:10.1073/pnas.0804812105. 18936492 PMC2575488

[8] Lloyd-Price J, Arze C, Ananthakrishnan AN, Schirmer M, Avila-Pacheco J, Poon TW, Andrews E, Ajami NJ, Bonham KS, Brislawn CJ, *et al*. Multi-omics of the gut microbial ecosystem in inflammatory bowel diseases. Nature. 2019;569(7758):655–662. doi:10.1038/s41586-019-1237-9. 31142855 PMC6650278

[9] Paramsothy S, Kamm MA, Kaakoush NO, Walsh AJ, van den Bogaerde J, Samuel D, Leong RWL, Connor S, Ng W, Paramsothy R, *et al*. Multidonor intensive faecal microbiota transplantation for active ulcerative colitis: a randomised placebo-controlled trial. Lancet. 2017;389(10075):1218–1228. doi:10.1016/s0140-6736(17)30182-4. 28214091

[10] Rossen NG, Fuentes S, van der Spek MJ, Tijssen JG, Hartman JHA, Duflou A, Löwenberg M, van den Brink GR, Mathus-Vliegen EMH, de Vos WM, *et al*. Findings from a randomized controlled trial of fecal transplantation for patients with ulcerative colitis. Gastroenterology. 2015;149(1):110–118.e4. doi:10.1053/j.gastro.2015.03.045. 25836986

[11] Costello SP, Hughes PA, Waters O, Bryant RV, Vincent AD, Blatchford P, Katsikeros R, Makanyanga J, Campaniello MA, Mavrangelos C, *et al*. Effect of fecal microbiota transplantation on 8-week remission in patients with ulcerative colitis: a randomized clinical trial. JAMA. 2019;321(2):156–164. doi:10.1001/jama.2018.20046. 30644982 PMC6439766

[12] Moayyedi P, Surette MG, Kim PT, Libertucci J, Wolfe M, Onischi C, Armstrong D, Marshall JK, Kassam Z, Reinisch W, *et al*. Fecal microbiota transplantation induces remission in patients with active ulcerative colitis in a randomized controlled trial. Gastroenterology. 2015;149(1):102–109.e6. doi:10.1053/j.gastro.2015.04.001. 25857665

[13] Sokol H, Landman C, Seksik P, Berard L, Montil M, Nion-Larmurier I, Bourrier A, Le Gall G, Lalande V, De Rougemont A, *et al*. Fecal microbiota transplantation to maintain remission in Crohn’s disease: a pilot randomized controlled study. Microbiome. 2020;8(1):12. doi:10.1186/s40168-020-0792-5. 32014035 PMC6998149

[14] Arthur JC, Perez-Chanona E, Mühlbauer M, Tomkovich S, Uronis JM, Fan TJ, Campbell BJ, Abujamel T, Dogan B, Rogers AB, *et al*. Intestinal inflammation targets cancer-inducing activity of the microbiota. Science (1979). 2012;338:120–123. doi:10.1126/science.1224820. PMC3645302 22903521

[15] Uronis JM, Mühlbauer M, Herfarth HH, Rubinas TC, Jones GS, Jobin C. Modulation of the intestinal microbiota alters colitis-associated colorectal cancer susceptibility. PLoS ONE. 2009;4(6):e6026–e6026. doi:10.1371/journal.pone.0006026. 19551144 PMC2696084

[16] Pratt M, Forbes JD, Knox NC, Van Domselaar G, Bernstein CN. Colorectal cancer screening in inflammatory bowel diseases - can characterization of gi microbiome signatures enhance neoplasia detection? Gastroenterology. 2022;162(5):1409–1423.e1. doi:10.1053/j.gastro.2021.12.287. 34998802

[17] Duboc H, Rajca S, Rainteau D, Benarous D, Maubert M-A, Quervain E, Thomas G, Barbu V, Humbert L, Despras G, *et al*. Connecting dysbiosis, bile-acid dysmetabolism and gut inflammation in inflammatory bowel diseases. Gut. 2013;62(4):531–539. doi:10.1136/gutjnl-2012-302578. 22993202

[18] Lavelle A, Sokol H. Gut microbiota-derived metabolites as key actors in inflammatory bowel disease. Nat Rev Gastroenterol Hepatol. 2020;17(4):223–237. doi:10.1038/s41575-019-0258-z. 32076145

[19] Ridlon JM, Wolf PG, Gaskins HR. Taurocholic acid metabolism by gut microbes and colon cancer. Gut Microbes. 2016;7(3):201–215. doi:10.1080/19490976.2016.1150414. 27003186 PMC4939921

[20] Ridlon JM, Devendran S, Alves JM, Doden H, Wolf PG, Pereira GV, Ly L, Volland A, Takei H, Nittono H, *et al*. The ‘ in vivo lifestyle’ of bile acid 7α-dehydroxylating bacteria: comparative genomics, metatranscriptomic, and bile acid metabolomics analysis of a defined microbial community in gnotobiotic mice. Gut Microbes. 2020;11(3):381–404. doi:10.1080/19490976.2019.1618173. 31177942 PMC7524365

[21] Vital M, Rud T, Rath S, Pieper DH, Schlüter D. Diversity of bacteria exhibiting bile acid-inducible 7α-dehydroxylation genes in the human gut. Comput Struct Biotechnol J. 2019;17:1016–1019. doi:10.1016/j.csbj.2019.07.012. 31428294 PMC6692061

[22] Heinken A, Ravcheev DA, Baldini F, Heirendt L, Fleming RMT, Thiele I. Systematic assessment of secondary bile acid metabolism in gut microbes reveals distinct metabolic capabilities in inflammatory bowel disease. Microbiome. 2019;7(1):75. doi:10.1186/s40168-019-0689-3. 31092280 PMC6521386

[23] Klimesova K, Kverka M, Zakostelska Z, Hudcovic T, Hrncir T, Stepankova R, Rossmann P, Ridl J, Kostovcik M, Mrazek J, *et al*. Altered gut microbiota promotes colitis-associated cancer in IL-1 receptor–associated kinase M–deficient mice. Inflamm Bowel Dis. 2013;19(6):1266–1277. doi:10.1097/MIB.0b013e318281330a. 23567778 PMC3744230

[24] Zhu W, Miyata N, Winter MG, Arenales A, Hughes ER, Spiga L, Kim J, Sifuentes-Dominguez L, Starokadomskyy P, Gopal P, *et al*. Editing of the gut microbiota reduces carcinogenesis in mouse models of colitis-associated colorectal cancer. J Exp Med. 2019;216(10):2378–2393. doi:10.1084/jem.20181939. 31358565 PMC6781011

[25] Arthur JC, Gharaibeh RZ, Mühlbauer M, Perez-Chanona E, Uronis JM, McCafferty J, Fodor AA, Jobin C. Microbial genomic analysis reveals the essential role of inflammation in bacteria-induced colorectal cancer. Nat Commun. 2014;5(1):4724. doi:10.1038/ncomms5724. 25182170 PMC4155410

[26] Nearing JT, Douglas GM, Hayes MG, MacDonald J, Desai DK, Allward N, Jones CMA, Wright RJ, Dhanani AS, Comeau AM, *et al*. Microbiome differential abundance methods produce different results across 38 datasets. Nat Commun. 2022;13(1):342. doi:10.1038/s41467-022-28034-z. 35039521 PMC8763921

[27] Richard ML, Liguori G, Lamas B, Brandi G, da Costa G, Hoffmann TW, Pierluigi Di Simone M, Calabrese C, Poggioli G, Langella P, *et al*. Mucosa-associated microbiota dysbiosis in colitis associated cancer. Gut Microbes. 2018;9(2):131–142. doi:10.1080/19490976.2017.1379637. 28914591 PMC5989788

[28] Sheridan PO, Duncan SH, Walker AW, Scott KP, Louis P, Flint HJ. Objections to the proposed reclassification of eubacterium rectale as Agathobacter rectalis. Int J Syst Evol Microbiol. 2016;66(5):2106. doi:10.1099/ijsem.0.000969. 26916277

[29] Rajilic-Stojanovic M, Shanahan F, Guarner F, de Vos WM. Phylogenetic analysis of dysbiosis in ulcerative colitis during remission. Inflamm Bowel Dis. 2013;19(3):481–488. doi:10.1097/MIB.0b013e31827fec6d. 23385241

[30] Pittayanon R, Lau JT, Leontiadis GI, Tse F, Yuan Y, Surette M, Moayyedi P. Differences in gut microbiota in patients with vs without inflammatory bowel diseases: a systematic review. Gastroenterology. 2020;158(4):930–946.e1. doi:10.1053/j.gastro.2019.11.294. 31812509

[31] Kolho KL, Korpela K, Jaakkola T, Pichai MVA, Zoetendal EG, Salonen A, de Vos WM. Fecal microbiota in pediatric inflammatory bowel disease and its relation to inflammation. Am J Gastroenterol Suppl. 2015;110(6):921–930. doi:10.1038/ajg.2015.149. 25986361

[32] Furusawa Y, Obata Y, Fukuda S, Endo TA, Nakato G, Takahashi D, Nakanishi Y, Uetake C, Kato K, Kato T, *et al*. Commensal microbe-derived butyrate induces the differentiation of colonic regulatory T cells. Nature. 2013;504(7480):446. doi:10.1038/nature12721. 24226770

[33] Fujimura KE, Sitarik AR, Havstad S, Lin DL, Levan S, Fadrosh D, Panzer AR, LaMere B, Rackaityte E, Lukacs NW, *et al*. Neonatal gut microbiota associates with childhood multisensitized atopy and T cell differentiation. Nat Med. 2016;22(10):1187–1191. doi:10.1038/nm.4176. 27618652 PMC5053876

[34] Jacobs JP, Goudarzi M, Singh N, Tong M, McHardy IH, Ruegger P, Asadourian M, Moon B-H, Ayson A, Borneman J, *et al*. A disease-associated microbial and metabolomics state in relatives of pediatric inflammatory bowel disease patients. Cell Mol Gastroenterol Hepatol. 2016;2(6):750–766. doi:10.1016/j.jcmgh.2016.06.004. 28174747 PMC5247316

[35] Png CW, Linden SK, Gilshenan KS, Zoetendal EG, McSweeney CS, Sly LI, McGuckin MA, Florin THJ. Mucolytic bacteria with increased prevalence in IBD mucosa augment in vitro utilization of mucin by other bacteria. Am J Gastroenterol Suppl. 2010;105(11):2420–2428. doi:10.1038/ajg.2010.281. 20648002

[36] Hall AB, Yassour M, Sauk J, Garner A, Jiang X, Arthur T, Lagoudas GK, Vatanen T, Fornelos N, Wilson R, *et al*. A novel ruminococcus gnavus clade enriched in inflammatory bowel disease patients. Genome Med. 2017;9(1):103. doi:10.1186/s13073-017-0490-5. 29183332 PMC5704459

[37] Gupta RB, Harpaz N, Itzkowitz S, Hossain S, Matula S, Kornbluth A, Bodian C, Ullman T. Histologic inflammation is a risk factor for progression to colorectal neoplasia in ulcerative colitis: a cohort study. Gastroenterology. 2007;133(4):1099–1105. doi:10.1053/j.gastro.2007.08.001. 17919486 PMC2175077

[38] Feng Q, Liang S, Jia H, Stadlmayr A, Tang L, Lan Z, Zhang D, Xia H, Xu X, Jie Z, *et al*. Gut microbiome development along the colorectal adenoma–carcinoma sequence. Nat Commun. 2015;6(1):6528. doi:10.1038/ncomms7528. 25758642

[39] Moschen AR, Gerner RR, Wang J, Klepsch V, Adolph T, Reider S, Hackl H, Pfister A, Schilling J, Moser P, *et al*. Lipocalin 2 protects from inflammation and tumorigenesis associated with gut microbiota alterations. Cell Host Microbe. 2016;19(4):455–469. doi:10.1016/j.chom.2016.03.007. 27078067

[40] Mahmoud R, Shah SC, ten Hove JR, Torres J, Mooiweer E, Castaneda D, Glass J, Elman J, Kumar A, Axelrad J, *et al*. No association between pseudopolyps and colorectal neoplasia in patients with inflammatory bowel diseases. Gastroenterology. 2019;156(5):1333–1344.e3. doi:10.1053/j.gastro.2018.11.067. 30529584 PMC7354096

[41] Sokol H, Leducq V, Aschard H, Pham H-P, Jegou S, Landman C, Cohen D, Liguori G, Bourrier A, Nion-Larmurier I, *et al*. Fungal microbiota dysbiosis in IBD. Gut. 2017;66(6):1039–1048. doi:10.1136/gutjnl-2015-310746. 26843508 PMC5532459

[42] Schmieder R, Edwards R. Quality control and preprocessing of metagenomic datasets. Bioinformatics. 2011;27(6):863–864. doi:10.1093/bioinformatics/btr026. 21278185 PMC3051327

[43] Martin M. Cutadapt removes adapter sequences from high-throughput sequencing reads. 2011. 2011;17:3. doi:10.14806/ej.17.1.200.

[44] Callahan BJ, McMurdie PJ, Rosen MJ, Han AW, Johnson AJA, Holmes SP. DADA2: high-resolution sample inference from Illumina amplicon data. Nat Methods. 2016;13(7):581. doi:10.1038/nmeth.3869. 27214047 PMC4927377

[45] Quast C, Pruesse E, Yilmaz P, Gerken J, Schweer T, Yarza P, Peplies J, Glöckner FO. The SILVA ribosomal RNA gene database project: improved data processing and web-based tools. Nucleic Acids Res. 2012;41(D1):D590–6. doi:10.1093/nar/gks1219. 23193283 PMC3531112

[46] R. R: a language and environment for statistical computing. 2018. http://www.r-project.org/

[47] McMurdie PJ, Holmes S, Watson M. phyloseq: an R package for reproducible interactive analysis and graphics of microbiome census data. PLoS ONE. 2013;8(4):e61217. doi:10.1371/journal.pone.0061217. 23630581 PMC3632530

[48] Callahan BJ, Sankaran K, Fukuyama JA, McMurdie PJ, Holmes SP. Bioconductor workflow for microbiome data analysis: from raw reads to community analyses. F1000Res. 2016;5:1492. doi:10.12688/f1000research.8986.2. 27508062 PMC4955027

[49] Oksanen J, Blanchet FG, Friendly M, Kindt R, Legendre P, McGlinn D, Minchin PR, O'Hara RB, Simpson GL, Solymos P, *et al*. vegan: community ecology package. *R package version 24-5*. Published Online First: 2017. https://cran.r-project.org/package=vegan

[50] Fernandes AD, Macklaim JM, Linn TG, Reid G, Gloor GB. ANOVA-Like Differential Expression (ALDEx) analysis for mixed population RNA-Seq. PLOS ONE. 2013;8(7):e67019. doi:10.1371/journal.pone.0067019. 23843979 PMC3699591

[51] Love MI, Huber W, Anders S. Moderated estimation of fold change and dispersion for RNA-seq data with DESeq2. Genome Biol. 2014;15(12):550. doi:10.1186/s13059-014-0550-8. 25516281 PMC4302049

[52] Wickham H. ggplot2: elegant graphics for data analysis. Springer New York. 2009.

[53] Kassambara A. ggpubr: “ggplot2” based publication ready plots. R package version 0.2.3. Published Online First: 2019. https://cran.r-project.org/package=ggpubr

[54] Holmes I, Harris K, Quince C, Gilbert JA. Dirichlet multinomial mixtures: generative models for microbial metagenomics. PLoS ONE. 2012;7(2):e30126. doi:10.1371/journal.pone.0030126. 22319561 PMC3272020

[55] Morgan M. DirichletMultinomial: dirichlet-multinomial mixture model machine learning for microbiome data. R Package Version 1260. 2019.

[56] Chong J, Xia J, Stegle O. MetaboAnalystR: an R package for flexible and reproducible analysis of metabolomics data. Bioinformatics. 2018;34(24):4313–4314. doi:10.1093/bioinformatics/bty528. 29955821 PMC6289126

[57] Morris JA, Gardner MJ. Statistics in medicine: calculating confidence intervals for relative risks (odds ratios) and standardised ratios and rates. Brit Med J (Clinical Research Ed). 1988;296(6632):1313–1316. doi:10.1136/bmj.296.6632.1313. PMC2545775 3133061

[58] Palarea-Albaladejo J, Martín-Fernández JA. zCompositions — r package for multivariate imputation of left-censored data under a compositional approach. Chemom Intell Lab Syst. 2015;143:85–96. doi:10.1016/j.chemolab.2015.02.019.

[59] Boogaart KG van den. compositions. R Package Lib CRAN. 2005;Version: 2.

