# Supplementary material for: Fecal microbiota and bile acids in IBD patients undergoing screening for colorectal cancer
Source: Gut Microbes. 2022 May 30;14(1):2078620. doi: 10.1080/19490976.2022.2078620 (PMC9176255; doi:10.1080/19490976.2022.2078620)
Supplement: Supplemental Material [file KGMI_A_2078620_SM3272.zip › Supplementary figures legends.docx]

**Figure S1 Results of differential abundance tests in IBD versus control**

a. Grid of genera detected by each differential abundance method comparing control subjects with CD. b. Grid of genera detected by each differential abundance method comparing control subjects with UC. CD – Crohn’s disease; UC – ulcerative colitis. P-values: * <0.05; ** <0.01; *** <0.001; **** <0.0001.

**Figure S2 Results of differential abundance tests in cancer and neoplasia versus no neoplasia in IBD**

a. Grid of genera detected by each differential abundance method comparing IBD patients without neoplasia and IBD patients with cancer. b. Grid of genera detected by each differential abundance method comparing UC patients without neoplasia and UC patients with neoplasia. c. Lachnospira abundance between UC patients with and without neoplasia. IBD – inflammatory bowel disease; UC – ulcerative colitis. P-values: * <0.05; ** <0.01; *** <0.001; **** <0.0001.

**Figure S3 Outputs of Dirichlet multinomial mixtures**

a. La Place model fit, with 3 clusters identified as an optimal fit. b. Parameters for the selected fit (this varies slightly between runs). c. Heatmap of the clusters on 20 re-runs of the Dirichlet multinomial mixture (DMM) fit, with samples as rows and runs as columns. 11 runs converged identically, while the remainder gave similar classification results.

**Figure S4 Sample storage and antibiotic use**

a. PCoA of samples with neoplasia stored in RNAlater® and frozen. b. Cross-tabulation of samples with neoplasia stored in RNAlater® and frozen based on cluster assignment. c. PCoA of samples evaluating effect of recent antibiotic use. d. PCoA evaluating the effect of sequencing run. PCoA – principle co-ordinate analysis.

**Figure S5 PCA of bile acid metabolites in IBD patients**

a. PCA of bile acids grouped by IBD neoplasia category. b. PCA of bile acids coloured by the log-transformed ratio of primary:secondary bile acids. c. PCA of bile acids coloured by the proportion of conjugated bile acids. d. PCA of bile acids coloured by the proportion of sulfated bile acids. e. Boxplots comparing bile acid conjugation by cluster. f. Heatmap of significant correlations between genera and bile acids (both clr-transformed). The side-bar indicates the cluster in which the mean abundance of the genus was highest. 1^o^ – primary; 2^o^ – secondary; PCA – principle component analysis.

**Figure S6 Bile acid profiles broken down by study group**

Boxplots comparing the proportion of the main 5 bile acids and bile acid conjugation in each cluster in in Control subjects, CD patients and UC patients. CA – cholic acid; CD – Crohn’s disease; CDCA – chenodeoxycholic acid; DCA – deoxycholic acid; LCA – lithocholic acid; UC – ulcerative colitis; UDCA – ursodeoxycholic acid. P-values: * <0.05; ** <0.01; *** <0.001; **** <0.0001.

**Figure S7 Ileal involvement in Crohn’s disease is associated with bile acid alterations**

Boxplots comparing the proportion of the main 5 bile acids and bile acid conjugation in CD patients with and without ileal involvement. CA – cholic acid; CDCA – chenodeoxycholic acid; DCA – deoxycholic acid; LCA – lithocholic acid; UDCA – ursodeoxycholic acid. P-values: * <0.05; ** <0.01; *** <0.001; **** <0.0001.
