# Supplementary figures and images for: Fecal microbiota and bile acids in IBD patients undergoing screening for colorectal cancer
Source: Gut Microbes. 2022 May 30;14(1):2078620. doi: 10.1080/19490976.2022.2078620 (PMC9176255; doi:10.1080/19490976.2022.2078620)

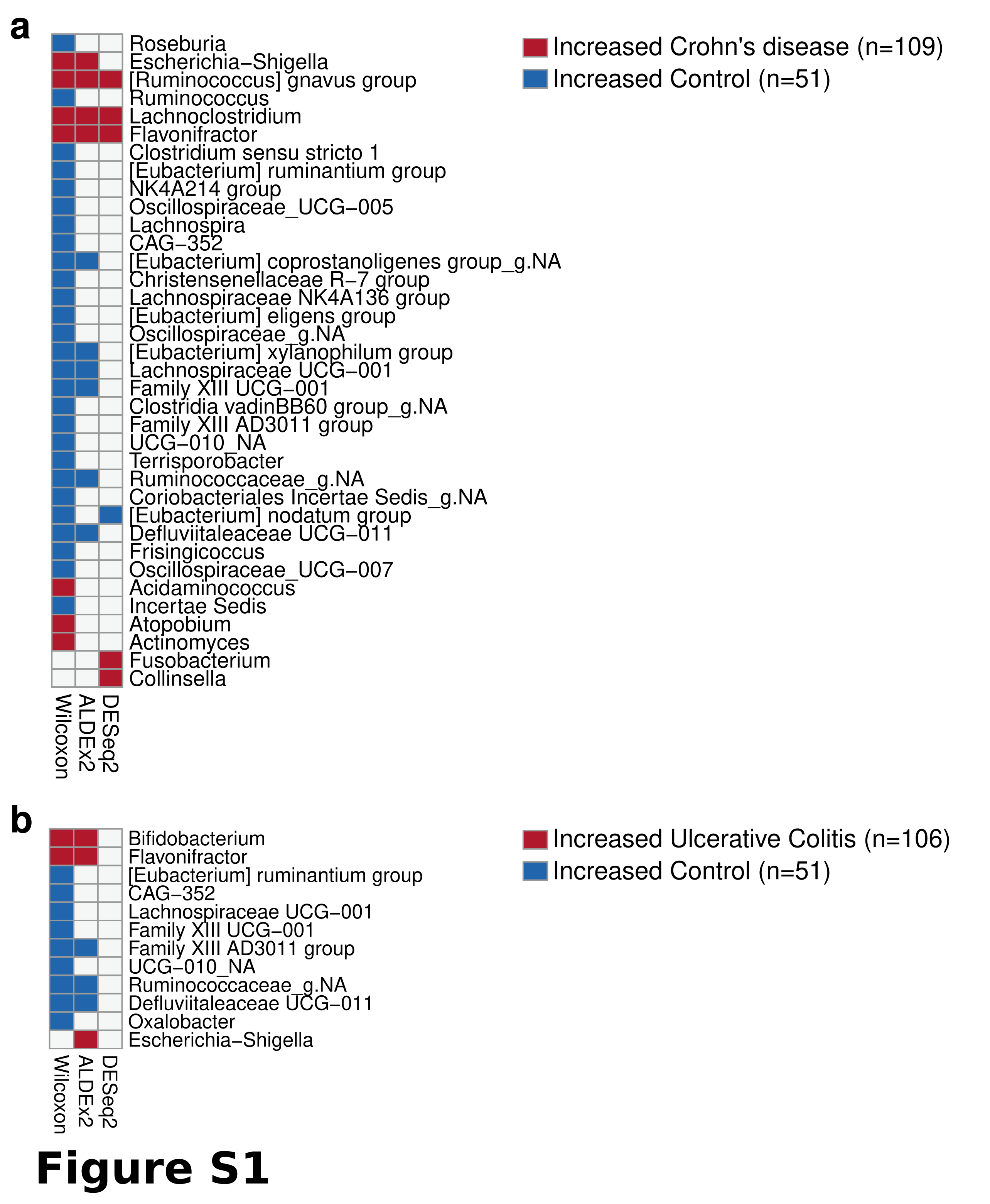

Supplement: Supplemental Material [file KGMI_A_2078620_SM3272.zip › J_FigureS1.png]

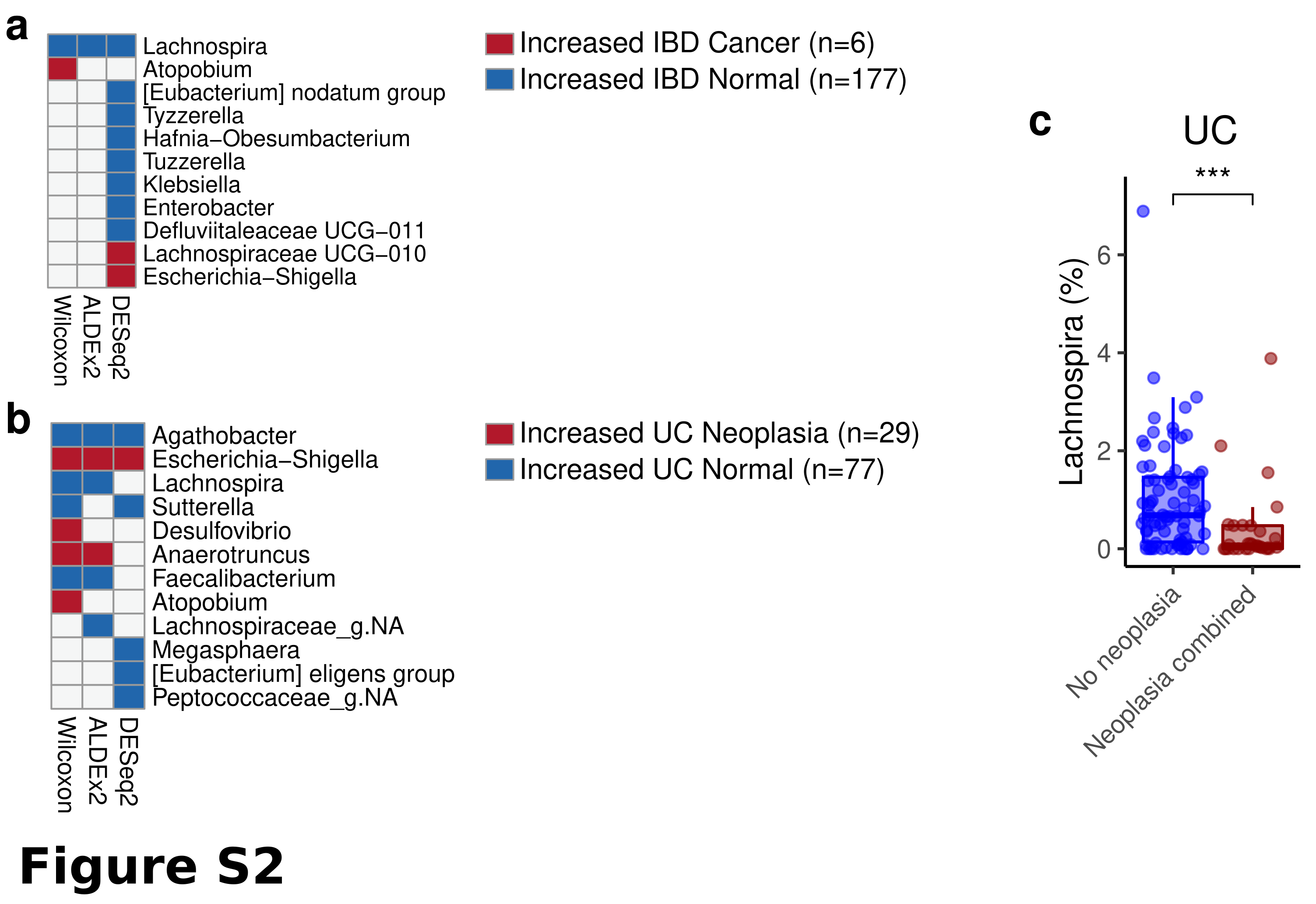

Supplement: Supplemental Material [file KGMI_A_2078620_SM3272.zip › K_FigureS2.png]

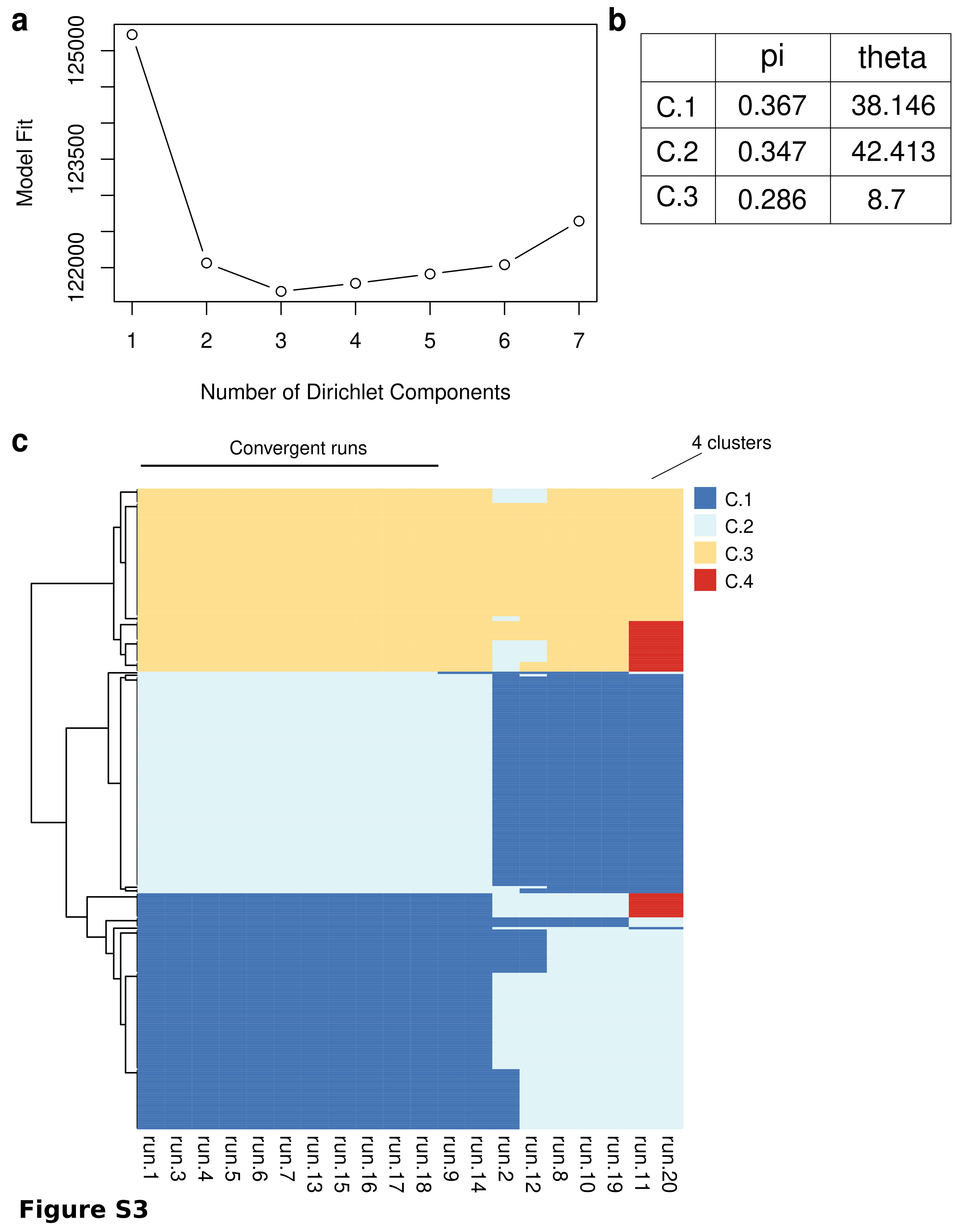

Supplement: Supplemental Material [file KGMI_A_2078620_SM3272.zip › L_FigureS3.png]

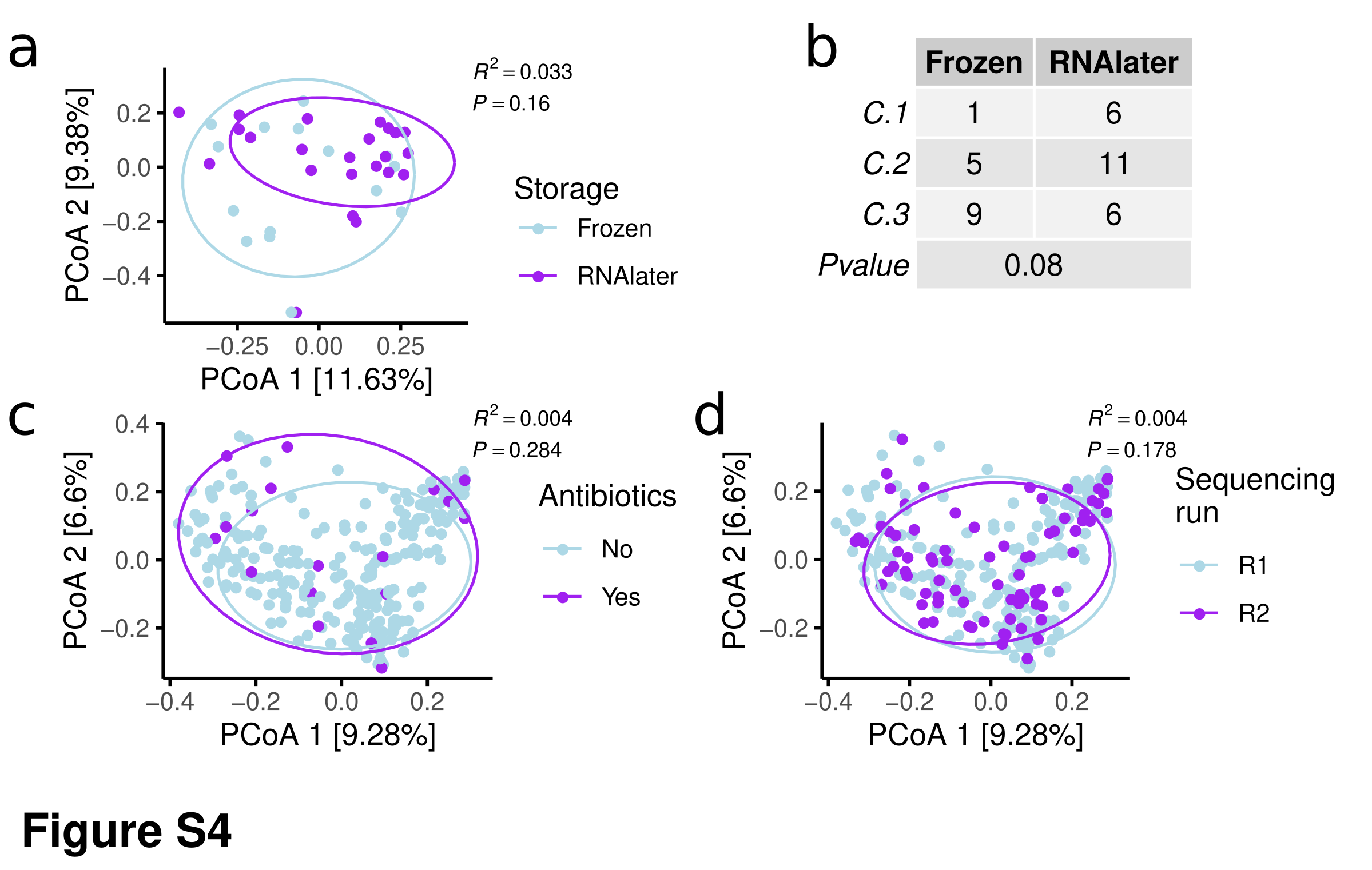

Supplement: Supplemental Material [file KGMI_A_2078620_SM3272.zip › M_FigureS4.png]

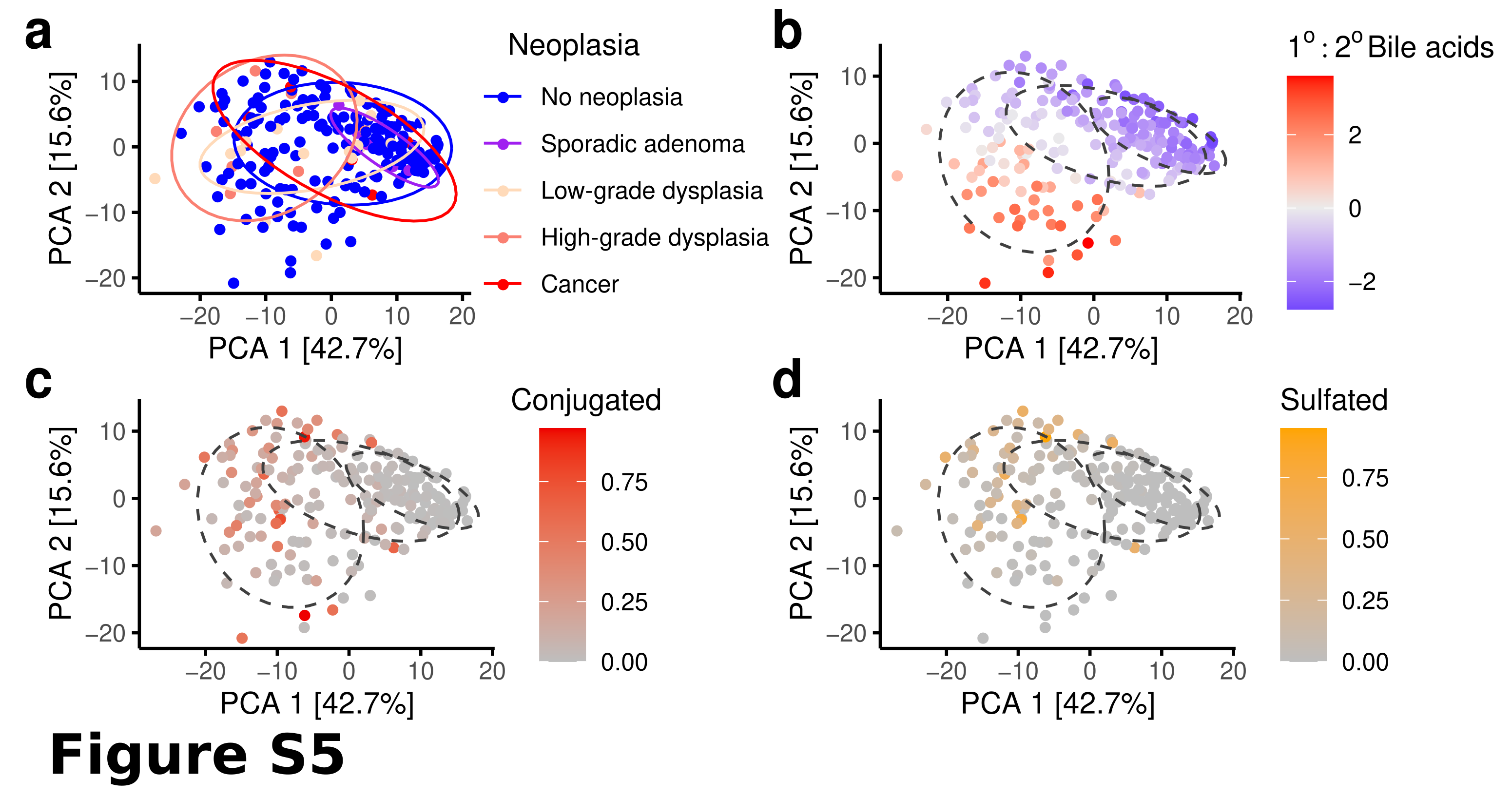

Supplement: Supplemental Material [file KGMI_A_2078620_SM3272.zip › N_FigureS5.png]

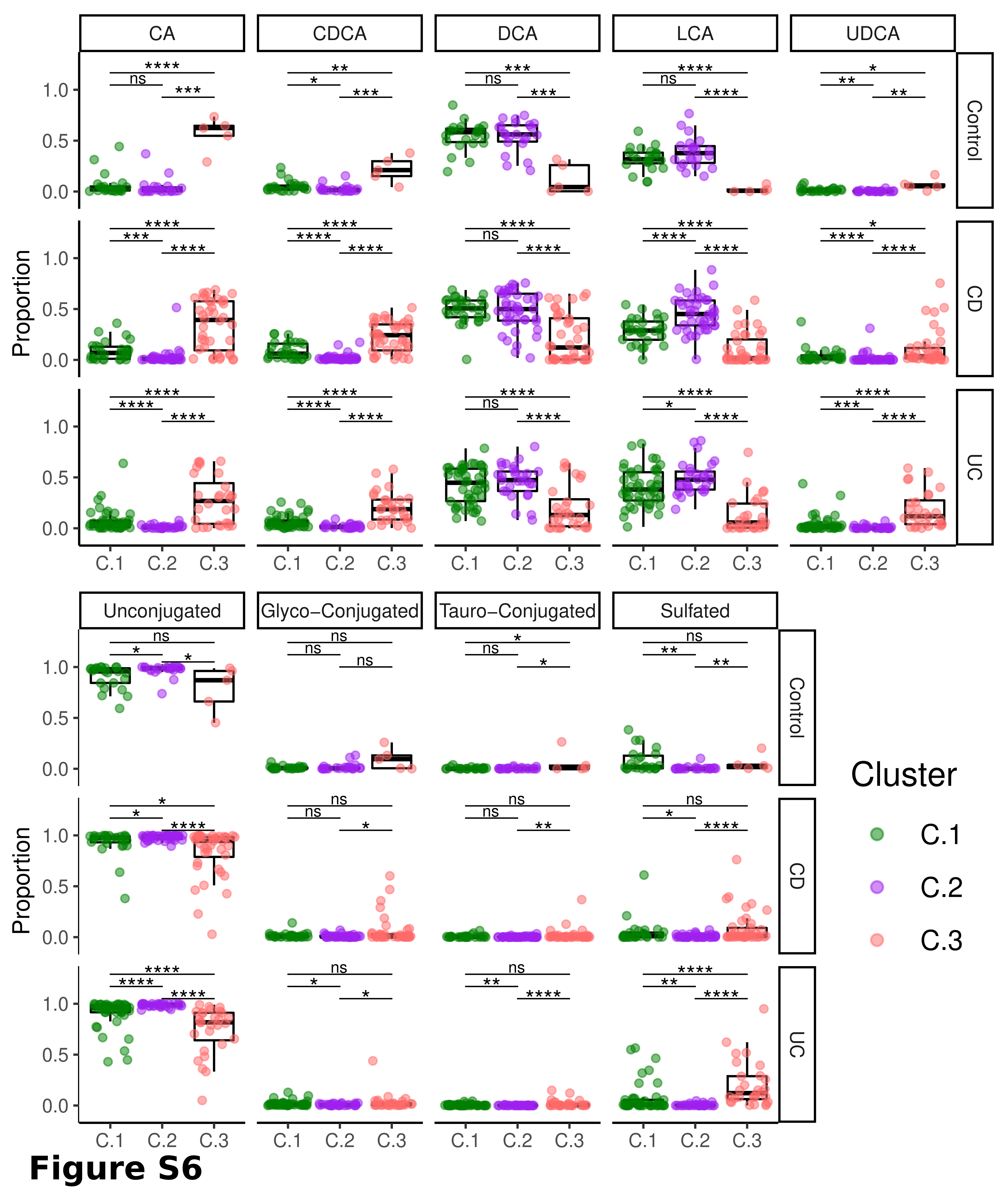

Supplement: Supplemental Material [file KGMI_A_2078620_SM3272.zip › O_FigureS6.png]

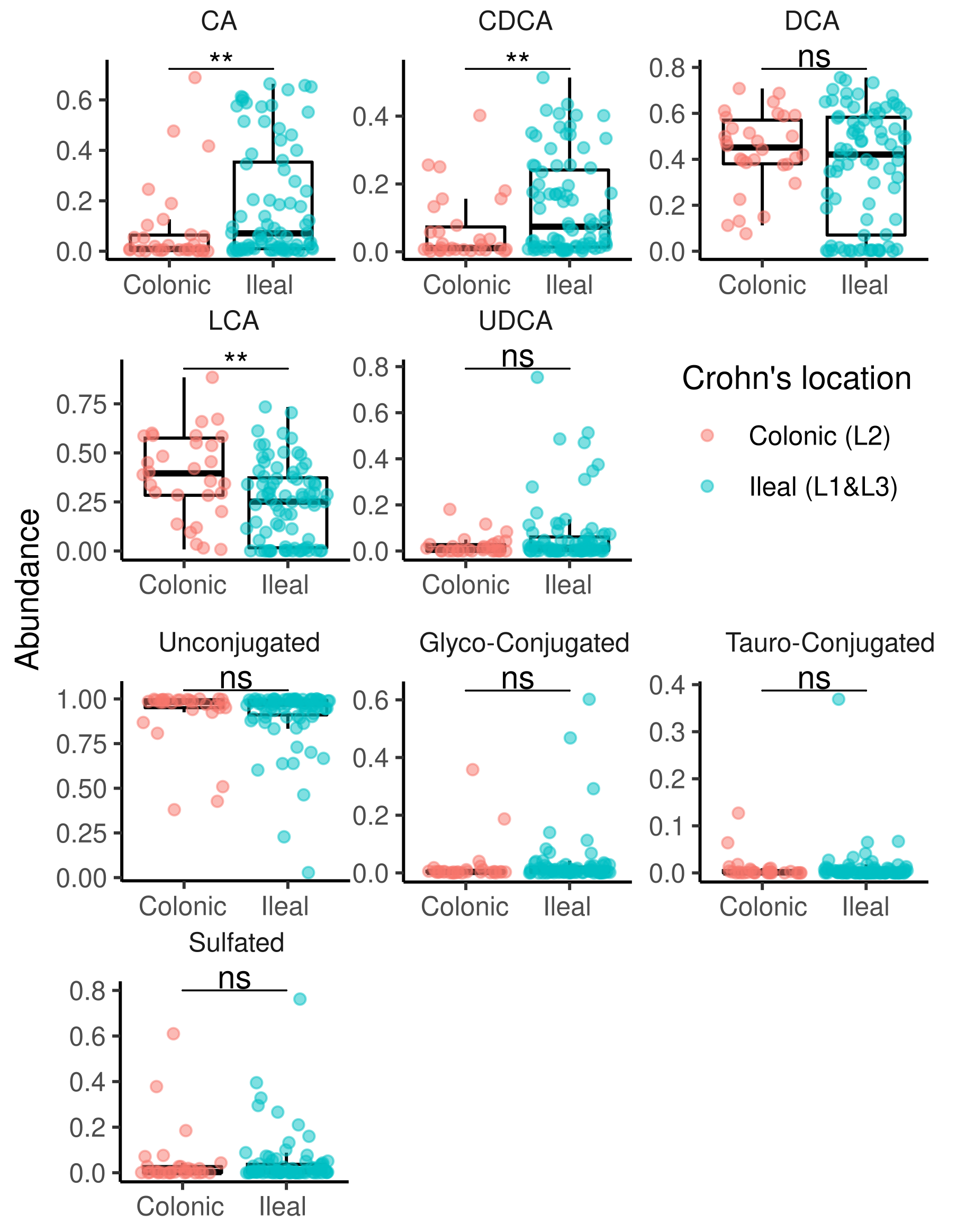

Supplement: Supplemental Material [file KGMI_A_2078620_SM3272.zip › P_FigureS7.png]
